# Multi-method evaluation of a 2-(1,3,4-thiadiazole-2-yl)pyrrolidine corrosion inhibitor for mild steel in HCl: combining gravimetric, electrochemical, and DFT approaches

**DOI:** 10.1038/s41598-023-36252-8

**Published:** 2023-06-16

**Authors:** Ahmed Al-Amiery, Wan Nor Roslam Wan Isahak, Waleed Khalid Al-Azzawi

**Affiliations:** 1 Department of Chemical and Process Engineering, Faculty of Engineering and Built Environment, Universiti Kebangsaan Malaysia, Bangi, Malaysia; 2 University of Technology-Iraq, Energy and Renewable Energies Technology Center, Bagdad, Iraq; 3 Al-Farahidi University, Baghdad, 10001 Iraq

**Keywords:** Materials science, Chemical physics, Quantum physics

## Abstract

The corrosion inhibition properties of 2-(1,3,4-thiadiazole-2-yl)pyrrolidine (2-TP) on mild steel in a 1 M HCl solution were investigated using weight loss, potentiodynamic polarization, electrochemical impedance spectroscopy (EIS) and open circuit potential (OCP) measurements. In addition, DFT calculations were performed on 2-TP. The polarization curves revealed that 2-TP is a mixed-type inhibitor. The results indicate that 2-TP is an effective inhibitor for mild steel corrosion in a 1.0 M HCl solution, with an inhibition efficiency of 94.6% at 0.5 mM 2-TP. The study also examined the impact of temperature, revealing that the inhibition efficiency increases with an increasing concentration of 2-TP and decreases with a rise in temperature. The adsorption of the inhibitor on the mild steel surface followed the Langmuir adsorption isotherm, and the free energy value indicated that the adsorption of 2-TP is a spontaneous process that involves both physical and chemical adsorption mechanisms. The DFT calculations showed that the adsorption of 2-TP on the mild steel surface is mainly through the interaction of the lone pair of electrons on the nitrogen atom of the thiadiazole ring with the metal surface. The results obtained from the weight loss, potentiodynamic polarization, EIS and OCP measurements were in good agreement with each other and confirmed the effectiveness of 2-TP as a corrosion inhibitor for mild steel in 1.0 M HCl solution. Overall, the study demonstrates the potential use of 2-TP as a corrosion inhibitor in acid environments.

## Introduction

**Figure 1:**
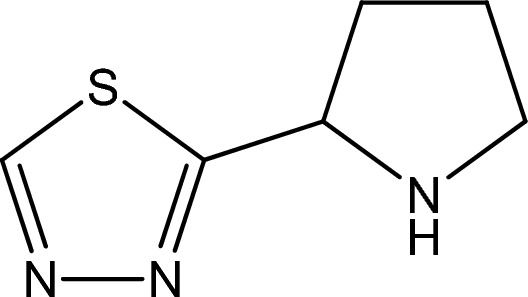
The chemical structure of 2-TP.

Thiadiazoles are a class of organic compounds with the formula (C_2_H_2_NH)_2_CS. They are heterocyclic compounds containing two nitrogen atoms and a sulfur atom in a ring system^1^. Thiadiazoles have various applications, including as fungicides, herbicides, insecticides, anti-inflammatory agents, and intermediate compounds in drug synthesis and the development of novel materials^2^. Some well-known thiadiazoles are metconazole and fenbuconazole. Our observations have led us to study the corrosion inhibition properties of 2-(1,3,4-thiadiazole-2-yl)pyrrolidine (2-TP). 2-TP was chosen as a corrosion inhibitor due to its heterocyclic structure, which includes a pyrrolidine functional group, three nitrogen atoms, and one sulfur atom in the ring. Its π electrons allow it to easily bond to mild steel (MS), reducing corrosion. It is commercially available or can be synthesized using green chemistry, resulting in a good yield. The mechanism of action of thiadiazoles as corrosion inhibitors is based on their ability to adsorb onto the metal surface and form a protective film. This film acts as a physical barrier, preventing corrosive species from reaching the metal surface and thus inhibiting the corrosion process. The adsorption process is influenced by factors such as the concentration of the inhibitor, pH, temperature, and the nature of the metal surface^3,4^. Mild steel is versatile and affordable, making it a popular choice for various industries^5,6^. It has low carbon content, making it easy to shape and weld, and its durability makes it ideal for construction and machinery^7–10^. Mild steel is also relatively cheap compared to other steels. Pickling is a metal surface treatment process that removes impurities, such as rust, scale, and oxidation, from the surface of metal. It is typically done by immersing the metal in a pickling solution, which is usually an acidic solution^11–13^. In industries, hydrochloric acid is commonly used as a pickling solution due to its high reactivity and ability to remove impurities efficiently. The concentration and temperature of the pickling solution are carefully monitored to ensure it does not damage the metal or corrode it^14,15^. Acid pickling is a process used to remove surface oxides and other impurities from steel by immersing it in an acidic solution. This process can lead to corrosion of the steel if not properly carried out. During the acid pickling process, the acid solution can attack the steel and dissolve some of the metal, leaving it vulnerable to corrosion. Additionally, if the pickling solution is not properly neutralized after the process, residual acid can remain on the steel and cause corrosion over time^16^. To prevent corrosion from acid pickling, it is important to use the appropriate acid concentration, temperature, and immersion time, and to properly neutralize the steel after the pickling process. Other factors such as the quality of the steel and the environment in which it is used can also affect its susceptibility to corrosion^17^. After the pickling process, the metal is rinsed with water, dried, and prepared for further processing or fabrication. Corrosion of substrates can be prevented or minimized through various methods, such as coating, galvanizing, electroplating, anodizing, using corrosion inhibitors, selecting corrosion-resistant materials, maintaining a controlled environment, and regular maintenance^18–20^. The molecular interaction between organic molecules and the metal surface is distributed or transmitted through chemical adsorption, resulting in the creation of a coordinated covalent bond. Physical adsorption involves Van der Waals and electrostatic interactions between the loaded organic molecules and the contaminated metal surface^21–23^. Physical adsorption is easily reversible and the organic molecules can be removed by simple washing or heating^23^. Chemical adsorption, on the other hand, is more permanent and the organic molecules become strongly bonded to the metal surface, making it more difficult to remove them^24,25^. Both physical and chemical adsorption play important roles in industrial processes such as catalysis, separation, and purification^26^. Understanding the differences between these two types of adsorption is critical in optimizing these processes^6,27–30^. Several types of thiadiazoles have been synthesized and tested as corrosion inhibitors. Some examples include 1,2,4-thiadiazole, 1,3,4-thiadiazole, 1,2,3-thiadiazole, and their derivatives. The inhibitive properties of these compounds depend on their chemical structure, and modifications can be made to enhance their effectiveness^31,32^. Studies have shown that thiadiazoles can effectively inhibit the corrosion of various metals, including steel, copper, and aluminum, in different environments such as acidic, neutral, and alkaline solutions. The inhibitive efficiency of thiadiazoles can be improved by combining them with other compounds, such as surfactants, and by using them in combination with other corrosion inhibitors^33^. Thiadiazoles have a wide range of applications in the field of corrosion inhibition, including in the oil and gas industry, the construction industry, and in the protection of cultural heritage sites. They are also used as additives in coatings and paints to improve their anti-corrosive properties^31–33^. Overall, thiadiazoles have shown promise as effective corrosion inhibitors for a variety of metals in different environments. Further research is needed to explore their potential in real-world applications and to optimize their inhibitive properties. The purpose of this paper is to examine the efficacy of 2-TP as a corrosion inhibitor for mild steel in a 1 M HCl solution. The impact of 2-TP (Fig. 1) at different concentrations was analyzed through weight loss, electrochemical impedance spectroscopy, potentiodynamic polarization, and adsorption isotherms. Density functional theory was also used to determine the corrosion inhibition efficiency of 2-TP. This method is cost-effective and efficient, offering a deeper understanding of the corrosion inhibition mechanism compared to traditional methods.

## Materials and methods

The base substrates for weight loss measurements and electrochemical corrosion experiments were mild steel samples purchased from the Company of Metal Samples. Specimens (Table 1) with a cubic area of 1 cm^2^ were used for PDP and EIS measurements, while specimens of 2.50 × 2.00 × 0.03 cm were employed in weight loss measurements. The specimens were prepared for each test by abrading with silicon carbide sheets (120, 600, and 1200) and washing with distilled water and acetone according to the standard technique, ASTM/G1-03^34^. The 1 M HCl assay solution was made with 37% analytical grade HCl and double-distilled water. The corrosion measurements were performed in non-stirring openair conditions with varying inhibitor concentrations.

**Table 1:** Chemical composition of metallic substrate (weight percentage).

Iron	Phosphorous	Sulphur	Aluminium	Silicon	Manganese	Carbon
Balance	0.09%	0.05%	0.01%	0.38%	0.05%	0.21%

### Weight loss techniques

The metallic substrate underwent testing with an untreated solution of 1 M HCl and a treated solution of 1 M HCl with a tested inhibitor at concentrations of 0.1, 0.2, 0.3, 0.4, 0.5, and 1.0 mM. The exposure durations were 1, 5, 10, 24, and 48 h at a temperature of 303 K. The effect of temperature was also studied by exposing the un-inhibited and inhibited solution to temperatures of 313, 323, and 333 K for 5 h, as specified by NACE TM0169/G31^35^. After testing, the coupons were removed and treated according to ASTM standard G1-03^34^. The mean mass loss was used to calculate the corrosion rate^35^, and the inhibitory performance (IE%) and surface coverage (θ) were determined using Eqs. (1–3)

1 $$C_{R } \left( {{\text{mg}}\,\,{\text{cm}}^{ - 2} \,\,{\text{h}}^{ - 1} } \right) = \frac{W}{at}$$

2 $$IE\% = \left[ {1 - \frac{{C_{R(i)} }}{{C_{Ro} }}} \right] \times 100$$

3 $$\theta = 1 - \frac{{C_{R\left( i \right)} }}{{C_{Ro} }}$$

where $W$ is the weight loss (mg), $a$ is the area (cm2), and $t$ is the exposure time (h).

### Electrochemical measurements

The electrochemical measurements were performed using a Gamry water-jacketed glass cell to measure the steady-state corrosion rate. The cell setup consisted of MS samples, a graphite rod, and a saturated calomel electrode (SCE) serving as the working, counter, and reference electrodes respectively. The analysis was conducted using a Gamry Device Potentiostat/Galvanostat/ZRA (REF 600) model and the accompanying software. The potentiodynamic polarization (PDP) and electrochemical impedance spectroscopy (EIS) measurements were obtained using the software^36^. The PDP curves were obtained by adjusting the potential between − 0.2 and + 0.2 V(SCE) over the corrosion potential with a scan rate of $0.5 \,\,{\text{mV/s}}$^5^. The EIS measurements were taken using alternating current signals with a $5.0 \,\,{\text{mV}}$ peak-to-peak amplitude at the corrosion potential and covering a frequency range of 100 kHz to 0.1 Hz. The impedance data was then fitted and simulated to the appropriate equivalent circuits using the Gamry Echem Analyst software. To ensure that the system reached a steady-state condition, the electrochemical corrosion tests were performed for 30 min after the working electrode was exposed to the environment. The experiments were performed three times, and the average values were recorded to confirm repeatability.

### DFT computations

In this study, quantum chemical calculations were performed using Gaussian 03 Revision C.01. The ground-state geometry was calculated with the 6-31G++ (d,p) valence and polarization basis set, and the optimization was done without symmetry constraints to reach a local minimum. The calculations were performed using the B3LYP approach, which combines the Becke three-parameter hybrid exchange functional with the Lee–Yang–Parr correlation functional^37^. The optimized geometry, HOMO and LUMO energies, and other physical parameters of the molecule were evaluated using this method. According to DFT-Koopman's theorem, the ionization potential is related to the HOMO energy (EHOMO), while the electron affinity is related to the LUMO energy (ELUMO). These values can be calculated using Eqs. 4 and 5^38^.

4 $$I = - E_{HOMO}$$

5 $$A = - E_{LOMO}$$

The examination of the Natural Bond Orbital (NBO) was performed to assess the distribution of electron densities, as electron density plays a crucial role in determining the parameters of chemical reactivity. The electronegativity (χ), hardness (η), and softness (σ) were calculated using Eqs. 6–8^39^.

6 $$\chi = \frac{I + A}{2}$$

7 $$\eta = \frac{I - A}{2}$$

8 $$\sigma = \eta^{ - 1}$$

The number of transferred electrons (ΔN) was calculated using a DFT approach using Eq. 9.

9 $$\Delta N = \frac{{\chi_{Fe} - \chi_{inh} }}{{2\left( {\eta_{Fe} + \eta_{inh} } \right)}}$$

This equation considers the absolute electronegativity of iron ($\chi_{Fe}$), which was found to be 7.0 eV, and the absolute electronegativity of the inhibitor molecule ($\chi_{inh}$). It also takes into account the absolute hardness of iron ($\eta_{Fe}$), which was found to be zero, and the absolute hardness of the inhibitor molecule ($\eta_{inh}$). So, Eq. 9 can be converted to Eq. 10^40^.

10 $$\Delta N = \frac{{7 - \chi_{inh} }}{{2\left( {\eta_{inh} } \right)}}$$

## Results and discussion

### Weight loss

#### Effect of the inhibitor concentration

**Figure 2:**
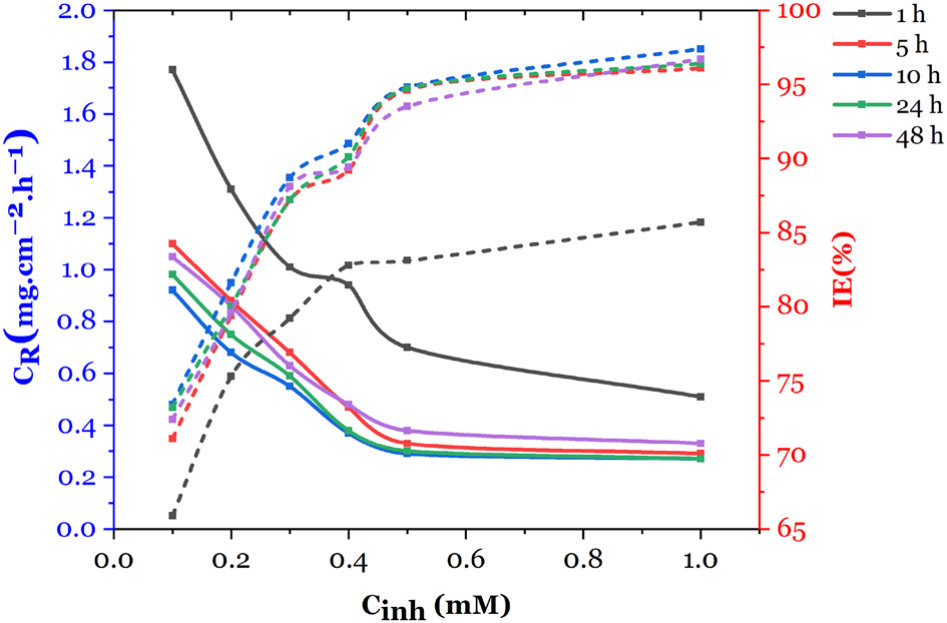
Influence of exposure time and concentration on the corrosion rate and inhibition efficiency of 2-TP on Mild Steel in 1 M HCl.

The protection of mild steel from corrosion was achieved using 2-TP, as shown in Fig. 2. The rate of corrosion (C_R_) and inhibition efficiency (IE%) were measured through weight loss tests at 303 K. The results showed that with an increase in 2-TP concentration, the CR decreased, resulting in improved inhibition due to the adsorption of more 2-TP molecules onto the mild steel, reducing its interaction with HCl. The highest inhibition efficiency of 94.6% was achieved at a 2-TP concentration of 0.5 mM. This is attributed to the electron-donating properties of the pyrrolidine and thiadiazole heterocyclic rings and the resonance effect of the thiadiazole ring and the inductive effect of the pyrrolidine ring, which improve the inhibitor's ability to transfer electron pairs to the unoccupied d-orbitals of the iron atoms on the mild steel surface, thereby controlling and preventing corrosion^41^. The duration of immersion also influenced the resistance of 2-TP against HCl (Fig. 2). The corrosion rate decreased over the first 10 h in 1 M HCl, reaching a maximum inhibition efficiency of 94.6%. 2-TP had the highest inhibition efficiency of 65.9% after 1 h, but after 10 h of immersion, the inhibition efficiency declined, reaching 94.8% at 0.5 mM and reducing to 88.1% after 48 h. The duration of immersion is a crucial factor in providing protection. The absorption of 2-TP molecules on the mild steel surface, which covers the area exposed to the HCl solution, is likely responsible for the decrease in corrosion rate and increase in inhibition efficiency as the 2-TP concentration increases.

2-TP has been demonstrated to be an effective corrosion inhibitor for mild steel in HCl solutions. The results showed that the inhibition efficiency increased with increasing 2-TP concentration and reached its maximum at 0.5 mM. The adsorption of 2-TP onto the mild steel surface was found to follow the Langmuir isotherm and was spontaneous and exothermic. The inhibitory effectiveness of 2-TP was compared to other nitrogen-based corrosion inhibitors for mild steel protection (Table 2) and demonstrated the highest inhibitory efficiency. 2-TP could be a promising alternative to current corrosion inhibitors, especially in industrial applications. Furthermore, 2-TP showed a stable and consistent inhibition performance in a wide range of immersion time values, temperatures, and corrosive environments. This indicates that it has a good versatility and adaptability, which is essential for industrial applications. Moreover, 2-TP has a low toxicity and environmental impact, making it a safer and more sustainable option for industrial applications compared to other nitrogen-based inhibitors^42^. To the best of our knowledge, there have been no reports of corrosion inhibitors suitable for HCl solutions. The inhibition efficiency of 0.5 mM 2-TP was found to be 94.6% at 303 K in a 1 M HCl solution, performing better than previously published inhibitors such as 2, 6, 8–12, 15–18, 20, 25, 26, 30, and 31. In the oil and gas industry, the bottom of the well is subjected to high temperatures, so a corrosion inhibitor must maintain its protective performance in corrosive media under such high temperatures.

**Table 2:** Comparison of 2-TP with other nitrogen-based corrosion inhibitors for protecting mild steel.

No	Inhibitor name	Alloy	Acid	IE(%)	References
1	2-(1,3,4-thiadiazole-2-yl)pyrrolidine (2-TP)	Mild steel	1 M HCl	94.6	–
2	*N*′-(2-(2-oxomethylpyrrol-1-yl)ethyl)piperidine	Mild steel	1 M HCl	91.9	^43^
3	2-Amino-4-phenyl-*N*-benzylidene-5-(1,2,4-triazol-1-yl)thiazole	Mild steel	1 M HCl	98.1	^44^
4	2-amino-4-phenylthiazole	Mild steel	1 M HCl	94.7	^45^
5	1-Amino-2-mercapto-5-(4-(pyrrol-1-yl)phenyl)-1,3,4-triazole	Mild steel	1 M HCl	96.3	^46^
6	*N*′-(2-hydroxybenzylidene)-2-(quinolin-8-yloxy)acetohydrazide	Mild steel	1 M HCl	93.4	^23^
7	3-(4-ethyl-5-mercapto-1,2,4-triazol-3-yl)-1- phenylpropanone	Mild steel	1 M HCl	97	^47^
8	4-ethyl-1-(4-oxo-4-phenylbutanoyl)thiosemicarbazide	Mild steel	1 M HCl	88.7	^48^
9	4-benzyl-1-(4-oxo-4-phenylbutanoyl)thiosemicarbazide	Mild steel	1 M HCl	92.5	^49^
10	4-chloro-2-((pyridin-2-ylimino)methyl)phenol	Mild steel	1 M HCl	92.8	^50^
11	2-*N*-phenylamino-5-(3-phenyl-3-oxo-1-propyl)-1,3,4-oxadiazole	Mild steel	1 M HCl	95.1	^51^
12	4-ethyl-1-(4-oxo-4-phenylbutanoyl)thiosemicarbazide	Mild steel	1 M HCl	88.7	^52^
13	Nonanedihydrazide	Mild steel	1 M HCl	97	^53^
14	4-pyrrol-1-yl-n-(2,5-dimethyl-pyrrol-1-yl)benzoylamine	Mild steel	1 M HCl	95.8	^54^
15	*N*′-(1-phenylethylidene)-4-(1H-pyrrol-1-yl)benzohydrazide	Mild steel	1 M HCl	94.5	^54^
16	5-((4-fluorobenzylidene)amino)-1,3,4-thiadiazole-2-thiol	Mild steel	1 M HCl	91	^55^
17	2-(5-amino-1,3,4-thiadiazol-2-yl)-5-nitrofuran	Mild steel	1 M HCl	83.2	^56^
18	Terephthalo- hydrazide	Mild steel	1 M HCl	96.4	^57^
19	Isophthalohydrazide	Mild steel	1 M HCl	97.2	^57^
20	*N*-(Naphthalen-1yl)-1-(4-pyridinyl)methanimine	Mild steel	1 M HCl	91.5	^58^
21	2-acetylthiophene thiosemicarbazon	Mild steel	1 M HCl	96	^59^
22	2-isonicotinoyl-*N*-phenylhydrazinecarbothioamide	Mild steel	1 M HCl	96.3	^60^
23	2-amino-5-(naphthalen-2-ylmethyl)-1,3,4-thiadiazole	Mild steel	1 M HCl	95.1	^61^
24	5-(4-(1H-pyrrol-yl)phenyl)-2-mercapto-1,3,4-oxadiazole	Mild steel	1 M HCl	95	^62^
25	*N*-(2,4-dihydroxytolueneylidene)-4-methylpyridin-2-amine	Mild steel	1 M HCl	93.7	^63^
26	*N*-methyl-2–1-5-methylthiophene-2-yl)ethylidene) hydrazine carbothioamide	Mild steel	1 M HCl	95.3	^64^
27	1-phenyl-2-(1-phenylethylidene)hydrazine	Mild steel	1 M HCl	83.8	^65^
28	1-(1-(4-methoxyphenyl)ethylidene)-2-phenylhydrazine	Mild steel	1 M HCl	95.1	^65^
29	2-(2,4-dimethoxybenzylidene)-Nphenylhydrazinecarbothioamide	Mild steel	1 M HCl	94.8	^66^
30	2-(5-amino-1,3,4-oxadiazol-2-yl)-5-nitrofuran	Mild steel	1 M HCl	79.4	^67^
31	8-piperazine-1-ylmethylumbelliferone	Mild steel	1 M HCl	93.4	^68^
32	2-((6-methyl-2-ketoquinoUne-3-yl)methylene) hydrazinecarbothioamide	Mild steel	1 M HCl	95.8	^69^
33	4-(6-methylcoumarin)acetohydrazide	Mild steel	1 M HCl	94.5	^70^
34	4-(Benzoimidazole-2-yl)pyridine	Mild steel	1 M HCl	93.8	^71^
35	5,5′-(1,4-phenylene)bis(*N*-phenyl-1,3,4-thiadiazol-2-amine)	Mild steel	1 M HCl	94	^72^
36	APT	Mild steel	1 M HCl	95	^73^
37	APT-2	Mild steel	1 M HCl	96	^73^
38	APT-4	Mild steel	1 M HCl	92	^73^
39	PAT	Mild steel	1 M HCl	98	^73^
40	*N* (-benzo[d]thiazol-2-yl)-1-(thiophene-2-yl) methanimine (BTTM)	Mild Steel	H_2_SO_4_		^74^
41	PMTTA	Mild steel	1 M HCl	92	^75^
42	PATT	Mild steel	1 M HCl	91	^75^
43	PMTA	Mild steel	1 M HCl	87	^75^

In conclusion, the study showed that 2-TP has the potential to be a highly effective corrosion inhibitor for mild steel protection in industrial applications. Its high inhibitory efficiency, adaptability, and low toxicity make it a promising alternative to current inhibitors. Further studies should be conducted to evaluate the long-term performance and practical application of 2-TP in various industrial settings.

#### Effect of the temperature

**Figure 3:**
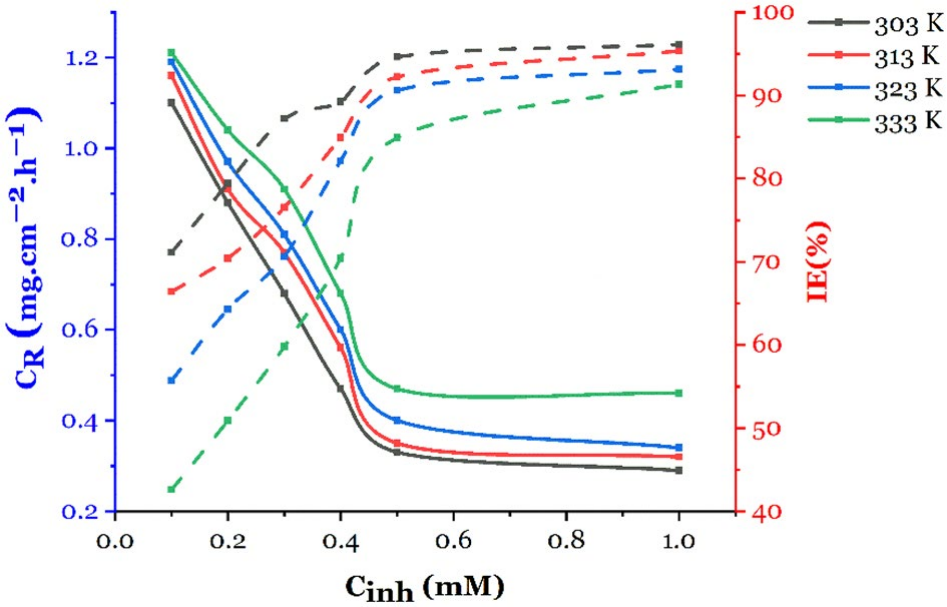
Influence of Temperature and concentration on the corrosion rate and inhibition efficiency of 2-TP on Mild Steel in 1 M HCl.

The impact of temperature on the performance of inhibitors is substantial. As the temperature increases from 323 to 333 K, the inhibition efficiency remains relatively constant. However, as shown in Fig. 3, when the temperature rises from 303 to 333 K, the efficiency of the 2-TP inhibitor drops from 94.6 to 84.9% at a concentration of 0.5 mM. This decrease can be attributed to the detachment of 2-TP molecules from the mild steel surface, causing a lack of protection against corrosion. As a result, the inhibition efficiency decreases as temperature increases.

Temperature also has a direct impact on the rate of chemical reactions, including corrosion reactions. At higher temperatures, the rate of reaction increases, leading to a faster rate of corrosion. Inhibitors slow down the rate of corrosion by forming a protective layer on the metal surface. At higher temperatures, this layer becomes less effective as the detachment of inhibitor molecules becomes more likely, causing a decrease in inhibition efficiency^72^. Moreover, elevated temperatures can lead to thermal degradation of the inhibitor molecules, reducing their ability to protect the metal surface. This results in a decrease in the inhibition efficiency as degraded molecules can no longer provide adequate protection against corrosion^76^. the decrease in 2-TP performance at elevated temperatures could be attributed to the thermal degradation of the inhibitor molecules, which leads to the loss of the protective film formed on the metal surface. The higher temperatures could also increase the rate of corrosion, leading to a faster breakdown of the protective film formed by the inhibitor. Additionally, the increased kinetic energy of the solution at higher temperatures could facilitate the adsorption of the inhibitor molecules onto other surfaces in the system, reducing the concentration of the inhibitor available to protect the metal surface.

In conclusion, temperature is a crucial factor that affects the effectiveness of inhibitors in controlling corrosion. It is important to consider the temperature conditions in which inhibitors will be used to ensure optimal performance and maximum protection against corrosion.

### Adsorption isotherm

Adsorption isotherms are useful for obtaining crucial information about the adsorption of inhibitor molecules on mild steel surfaces. The surface coverage (θ) was calculated through weight loss measurements and was used to identify the most suitable isotherm. To determine if the 2-TP molecules were physically or chemically attached to the mild steel surface, various adsorption isotherms, such as Temkin, Freundlich, and Langmuir, were analyzed. The Langmuir isotherm was considered the most appropriate model as it had a linear regression coefficient close to one. The Langmuir isotherm can be expressed using Eq. 11.

11 $$\frac{C}{\theta } = \frac{1}{{K_{ads} }} + C$$

where $C$ is the inhibitor concentration and $K_{ads}$, is the Langmuir constant.

The Freundlich adsorption isotherm is commonly used to describe adsorption on surfaces with varying active sites, energies, and surface heterogeneity. It defines the exponential distribution of these factors. Equation 12 represents the Freundlich adsorption isotherm.

12 $$log\theta = logK_{F} + logC$$

The Temkin adsorption isotherm takes into account the indirect interactions between adsorbate molecules and the adsorption process. This is particularly important when the heat of adsorption of molecules in the layer is inversely proportional to the surface coverage. However, this model is only valid for a limited range of concentrations. Equation 13 shows the Temkin isotherm.

13 $$e^{ - 2a\theta } = KC$$

**Figure 4:**
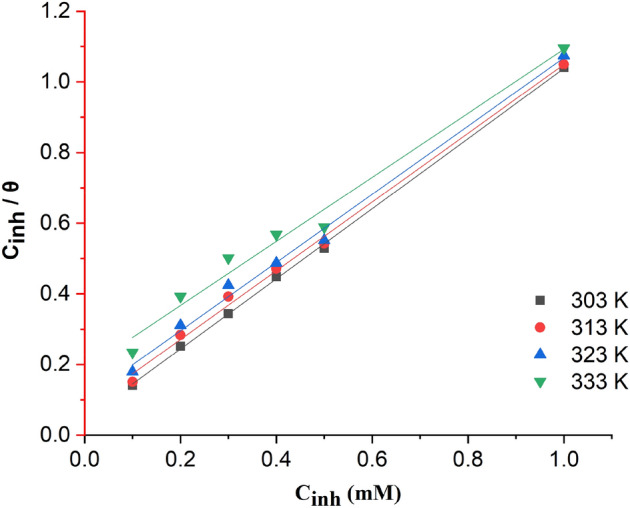
2-TP plot of Langmuir adsorption isotherm.

**Figure 5:**
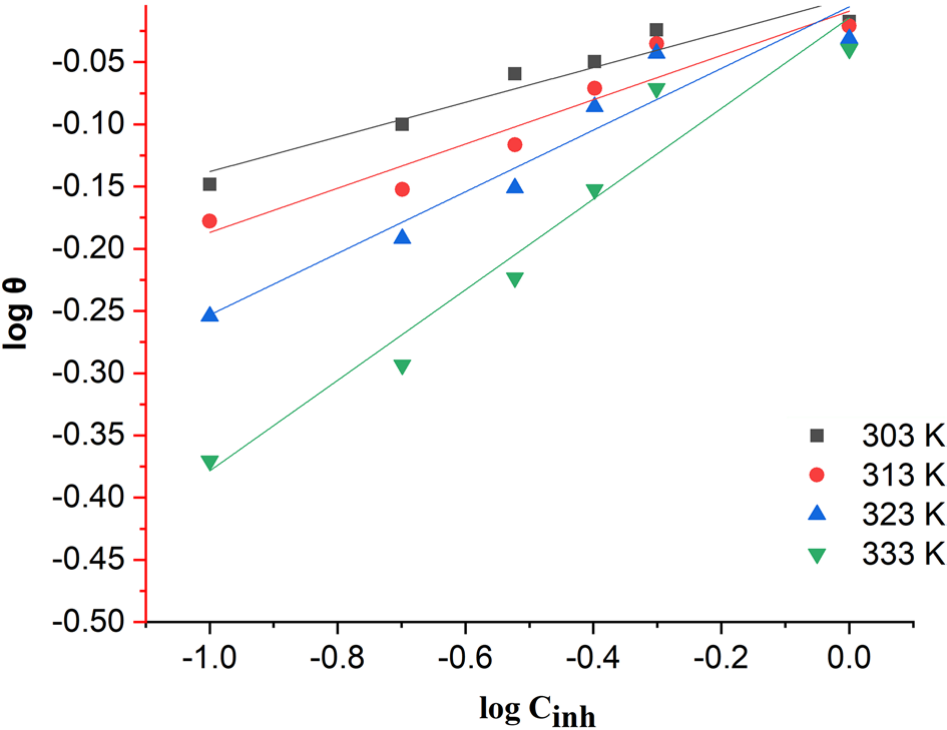
2-TP plot of Freundlich adsorption isotherm.

**Figure 6:**
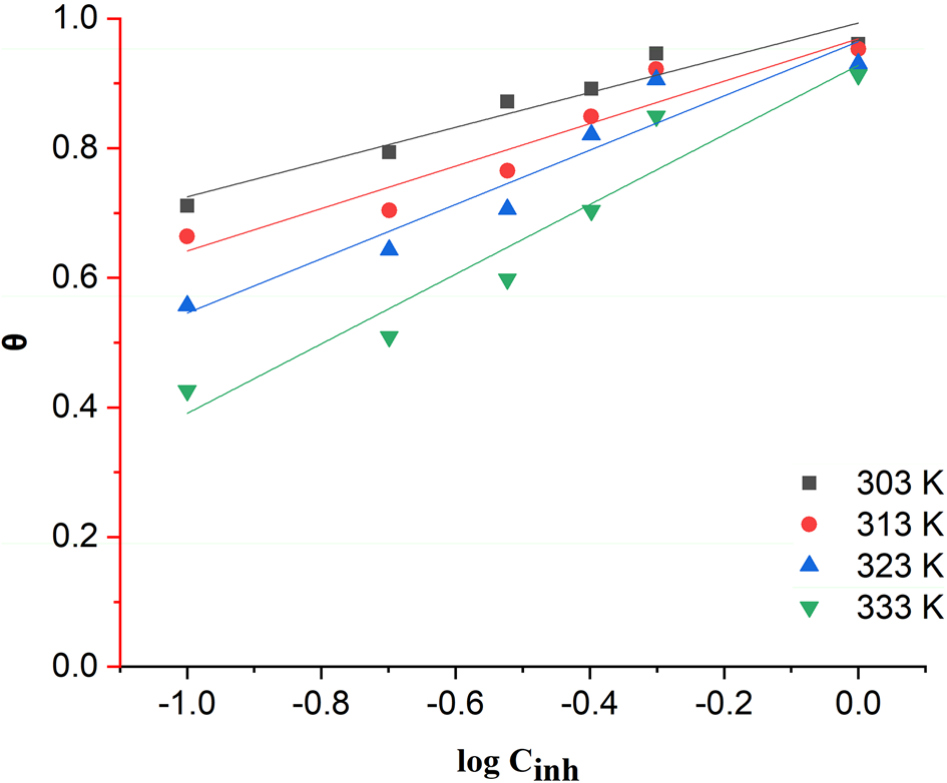
2-TP plot of Temkin adsorption isotherm.

The isotherm and thermodynamic analyses provided sufficient information to explain the inhibition potential and adsorption mechanism of 2-TP molecules at the interface between hydrochloric acid solution and mild steel. The estimated parameters obtained from the Langmuir (Fig. 4), Freundlich (Fig. 5), and Temkin (Fig. 6) isotherm graphs at different temperatures are presented in Table 3. The results indicate that the Langmuir isotherm model provided the best fit for the adsorption of 2-TP molecules on the surface of mild steel at all studied temperatures and times. This was confirmed by high R2 values and a slope close to unity. The Langmuir isotherm assumes that the adsorption of inhibitor molecules onto the mild steel surface is a monolayer process and that the process is energetically favorable^77^. The Langmuir constant provides valuable information about the interaction between the inhibitor molecules and the mild steel surface, as well as the mild steel surface's ability to adsorb the inhibitor molecules. Furthermore, the Langmuir isotherm can be utilized to determine the maximum adsorption capacity of the mild steel surface for the inhibitor molecules. The maximum adsorption capacity can then be used to calculate the optimal amount of inhibitor molecules needed to prevent corrosion of the mild steel surface^77^. Overall, the Langmuir isotherm provides valuable insights into the adsorption behavior of the inhibitor molecules on the mild steel surface and can guide the optimization of the inhibitor treatment process. Based on Table 3, the Langmuir isotherm was the most appropriate fit for all the temperatures tested. At 303 K, the regression coefficient (R^2^) was calculated to be 0.999 with a computed slope value of 0.99212 ± 0.01206 and an intercept value of 0.04564 ± 0.00613. Figure 4 illustrates the Langmuir isothermal plot between C/θ and C for the temperature range of 303–333 K.

**Table 3:** Adsorption parameters for inhibition of mild steel in treated solution at different temperature.

Parameter	Temperature			
	303 K	313 K	323 K	333 K
Langmuir				
Slop	0.992 ± 0.012	0.972 ± 7.073	0.965 ± 9.51	0.905 ± 7.977
Intercept	0.045 ± 0.006	0.076 ± 4.303	0.102 ± 5.791	0.186 ± 4.853
R^2^	0.999	1	1	1
Freundlich				
Slop	0.139 ± 0.019	0.177 ± 0.026	0.247 ± 0.034	0.364 ± 0.04
Intercept	0.001 ± 0.011	− 0.009 ± 0.015	− 0.005 ± 0.02	− 0.014 ± 0.025
R^2^	0.92688	0.9203	0.92781	0.94576
Temkin				
Slop	0.268 ± 0.035	0.327 ± 0.050	0.418 ± 0.060	0.537 ± 0.073
Intercept	0.993 ± 0.020	0.968 ± 0.028	0.964 ± 0.035	0.928 ± 0.042
R^2^	0.93621	0.91453	0.92201	0.93045

The relationship between the adsorbent and adsorbate is expressed by the adsorption constant ($K_{ads}$). A higher $K_{ads}$ indicates improved adsorption and thus improved inhibition^78^. The relationship between the adsorption free Gibbs energy and the adsorption equilibrium constant can be represented by Eq. 14.

14 $$\Delta G_{ads}^{o} = - RT{ }ln\left( {55.5K_{ads} } \right)$$

where T is the temperature, R is the gas constant, and 55.5 is the water content measurement. The “$K_{ads}$” constant was added to the calculation above to produce the “$\Delta G_{ads}^{o}$” value.

Equation (12) demonstrates that the free Gibbs energy of adsorption is proportional to the natural logarithm of the adsorption equilibrium constant, $K_{ads}$. The higher the $K_{ads}$, the lower the free Gibbs energy of adsorption and vice versa. In simpler terms, a low free Gibbs energy of adsorption means that the adsorbent and the adsorbate have a strong bond, leading to a high adsorption rate.

Therefore, an increase in $K_{ads}$ leads to a decrease in the adsorption free Gibbs energy, resulting in better adsorption and improved inhibition. Hence, $K_{ads}$ plays a critical role in determining the efficiency of the adsorption process and the inhibition of the adsorbate. In conclusion, the adsorption equilibrium constant, $K_{ads}$, is a crucial parameter that can predict the efficiency of the adsorption process and the inhibition of the adsorbate. The relationship between $K_{ads}$ and the adsorption free Gibbs energy, as demonstrated by Eq. (12), emphasizes the importance of $K_{ads}$ in comprehending the adsorption behavior of a system^79^. A negative value of the adsorption $\Delta G_{ads}^{o}$ indicates spontaneity, and the inhibitor molecules are absorbed onto mild steel. A $\Delta G_{ads}^{o}$ value of ≤ − 20kJmol^−1^ indicates physical adsorption of the inhibitor molecule to the mild steel surface. However, a significantly negative adsorption free energy value of ≥ − 40 kJmol^−1^ suggests chemical adsorption, with the formation of coordination interactions between the 2-TP molecules and iron atoms on the mild steel surface. The estimated value of $\Delta G_{ads}^{o}$ being − 31.4 kJmol^−1^ indicates a mixed-mode interaction, incorporating both physical and chemical adsorption. The calculated $\Delta G_{ads}^{o}$ value indicates that the adsorption process of inhibitor molecules onto the mild steel surface is spontaneous and energetically favorable. The negative value shows that the system releases energy when the inhibitor molecules are adsorbed, leading to a decrease in the overall energy of the system. The magnitude of $\Delta G_{ads}^{o}$ also reveals the type of adsorption that takes place. In physical adsorption, the process is driven by van der Waals forces, dipole–dipole interactions, and hydrogen bonding. The inhibitor molecules are attached to the mild steel surface through weak non-covalent interactions and is usually indicated by a $\Delta G_{ads}^{o}$ value of less than − 20 kJmol^−1^. In chemical adsorption, the process involves covalent bonding between the inhibitor molecules and the mild steel surface iron atoms. This type of adsorption is usually indicated by a $\Delta G_{ads}^{o}$ value of less than − 40 kJ/mol, which represents the energy required to break the chemical bonds. The calculated $\Delta G_{ads}^{o}$ value of ≥ − 31.4 kJmol^−1^ suggests a mixed-mode interaction between the inhibitor molecules and the mild steel surface. This indicates that both physical and chemical adsorption are taking place simultaneously, with the inhibitor molecules held to the surface through both weak non-covalent interactions and covalent bonding. Overall, the results suggest that the adsorption of inhibitor molecules onto the mild steel surface is energetically favorable and that mixed-mode interactions are occurring between the inhibitor molecules and the surface.

Thermodynamics studies.

### Thermodynamics studies

**Figure 7:**
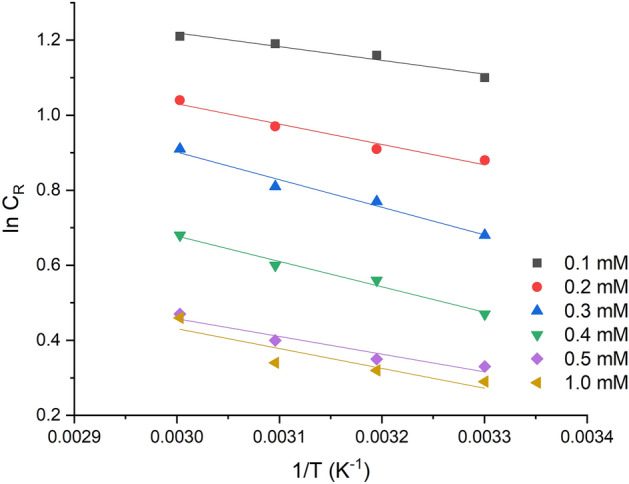
Arrhenius plot of ln CR against 1/T for mild steel with the addition of various of 2-TP after 5 h of exposure time.

Figure 7 in the study presents an Arrhenius plot used to estimate the activation energy (Ea) after 5 h of mild steel immersion in 1 M HCl, in the presence of various concentrations of 2-TP. The values of Ea were estimated to be within the range of 22.56–26.85 kJ/mol for all 2-TP concentrations. The values of activation energies (E_a_) below 80 kJ/mol indicate physical adsorption, while values above 80 kJ/mol indicate chemical adsorption^80,81^. Therefore, it was concluded that the interaction of 2-TP molecules leading to mild steel surface adsorption followed a chemical-physical mechanism.

**Figure 8:**
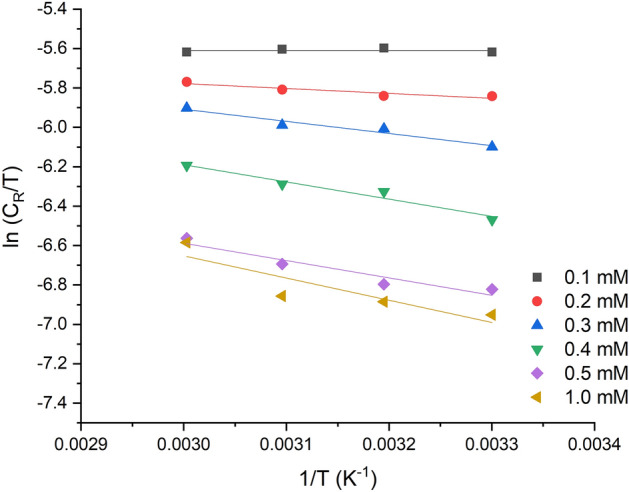
Transition state plot of In (CR/T) against 1/T for mild steel with the addition of various of 2-TP after 5 h of exposure time.

In Fig. 8, the transition state plot for the control and inhibited system after 5 h of the corrosion process is presented. The negative values of the enthalpy changes (ΔHAds) for different concentrations of 2-TP indicated exothermic behavior^82^, with a range of − 36.83 to − 43.11 kJ/mol across the test temperature (303–333 K), suggesting a chemisorption-physisorption mechanism. Negative values of entropy ΔSa revealed a decrease in disorderliness. The adsorption free energy (ΔGa) ranged between − 31.4 and − 35.72 kJ/mol. The negative ΔGa values indicate the spontaneous and feasible nature of the adsorption process. These values at 303–333 K showed good interaction between the 2-TP molecules and the mild steel surface, suggesting strong adsorption of 2-TP on mild steel^83^. Therefore, it was concluded that the adsorption of 2-TP molecules on the mild steel surface followed a physical and chemical adsorption mechanism.

### Electrochemical corrosion measurements

#### EIS

**Figure 9:**
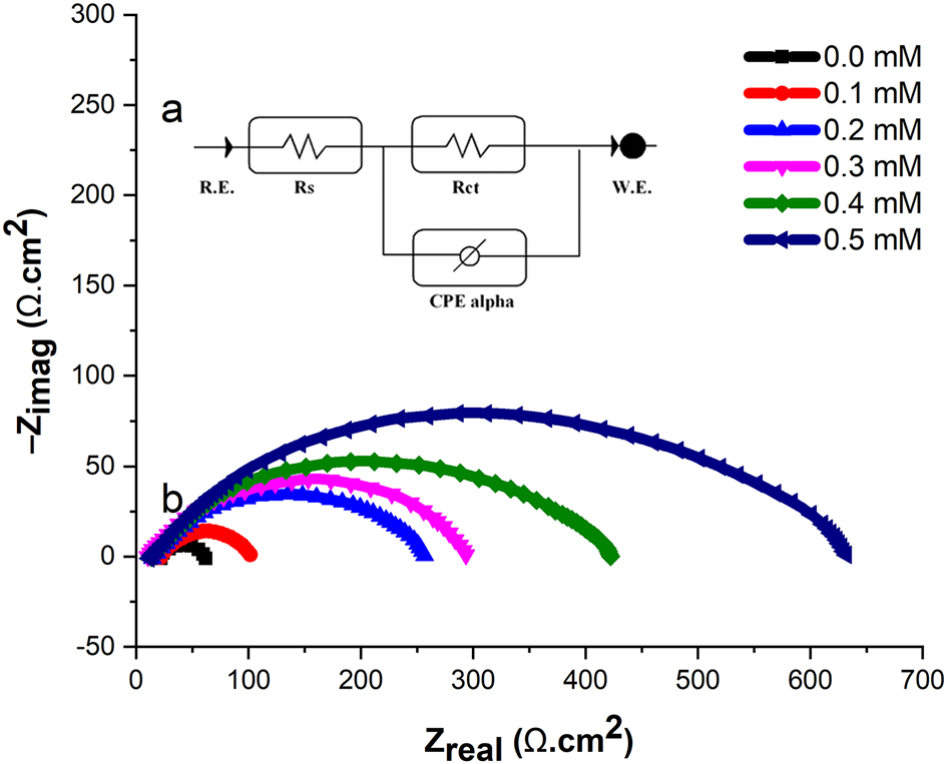
(**a**) Nyquist plots for mild steel in untreated and treated solutions at 303 K, (**b**) Equivalent circuit model utilised to fit impedance data in in untreated and treated solutions.

The frequency range used in the electrochemical impedance spectroscopy (EIS) measurements can provide important information about the system being studied. For example, the high-frequency region can provide information about the surface capacitance and double-layer structure, while the low-frequency region can provide information about charge transfer processes and mass transport limitations. The Gamry Analyst software was used to analyze the EIS experimental data, including the calculation of *CPE*, *R*_*s*_, *R*_*ct*_, and *C*_*dl*_ for mild steel in 1.0 M HCl solution^84^. Table 4 presents a comparison of the *CPE* of mild steel at different inhibitor concentrations at 303 K. The R_ct_ value increases with an increasing inhibitor concentration, indicating that the corrosion inhibitors adsorb on the mild steel sample surface, forming a protective layer that slows down corrosion. At a concentration of 0.5 mM, the inhibition efficiency reached 89.39% due to the elevated R_ct_ value. The addition of the corrosion inhibitor significantly improves the overall impedance of the mild steel sample, as illustrated in Fig. 9. The inhibition efficiency was calculated from the charge transfer resistance using Eq. (15).

15 $$IE\% = \frac{{R_{inh} - R_{uinh} }}{{R_{inh} }} \times 100$$

**Table 4:** CPE data for mild steel in untreated and treated solutions at 303 K.

$C_{inh} \left( {{\text{mM}}} \right)$	$CPE_{dl}$	$C_{dl}$(µF cm^−2^)	$R_{ct}$(Ω cm^2^)	$R_{s}$(Ω cm^2^	IE (%)	
0.0	$Y_{o}$(µS s^−α^ e^−2^)	$\alpha$	98	0.0843	2.125	0.0
0.1	917	0.79	67	0.7595	0.747	64.39
0.2	528	0.73	58	0.7684	0.382	70.43
0.3	486	0.72	48	0.7739	0.394	72.63
0.4	469	0.82	40	0.8532	0.547	84.57
0.5	387	0.76	34	0.8837	0.418	89.39

The Nyquist plots show that the addition of 2-TP increases the diameter of the capacitive loop in mild steel compared to when it is immersed in 1 M HCl without 2-TP. The diameter of the Nyquist plot is a reflection of the polarization resistance (Rp)^85^. The larger diameter of the Nyquist plot in the presence of 2-TP signifies a higher polarization resistance, which implies a reduced corrosion rate. This is verified by the results in Table 4, which indicate that the polarization resistance (*Rp*) is significantly higher in the presence of 2-TP compared to 1 M HCl without 2-TP. This indicates that 2-TP has effectively inhibited the corrosion of mild steel in the test solution^86^. The electrolytic resistance (*Rs*) values are also higher in the presence of 2-TP, consistent with the increased polarization resistance. This elevated electrolytic resistance is caused by the formation of a protective film on the surface of mild steel, which acts as a barrier to stop the corrosion reaction^87,88^. In conclusion, the results of the EIS analysis demonstrate that the presence of 2-TP has a significant impact on the corrosion behavior of mild steel in the test solution. The 2-TP has effectively reduced the corrosion rate of mild steel by increasing the polarization resistance and forming a protective film on the surface of the metal. This demonstrates the potential of 2-TP as an effective corrosion inhibitor for mild steel in harsh environments.

**Figure 10:**
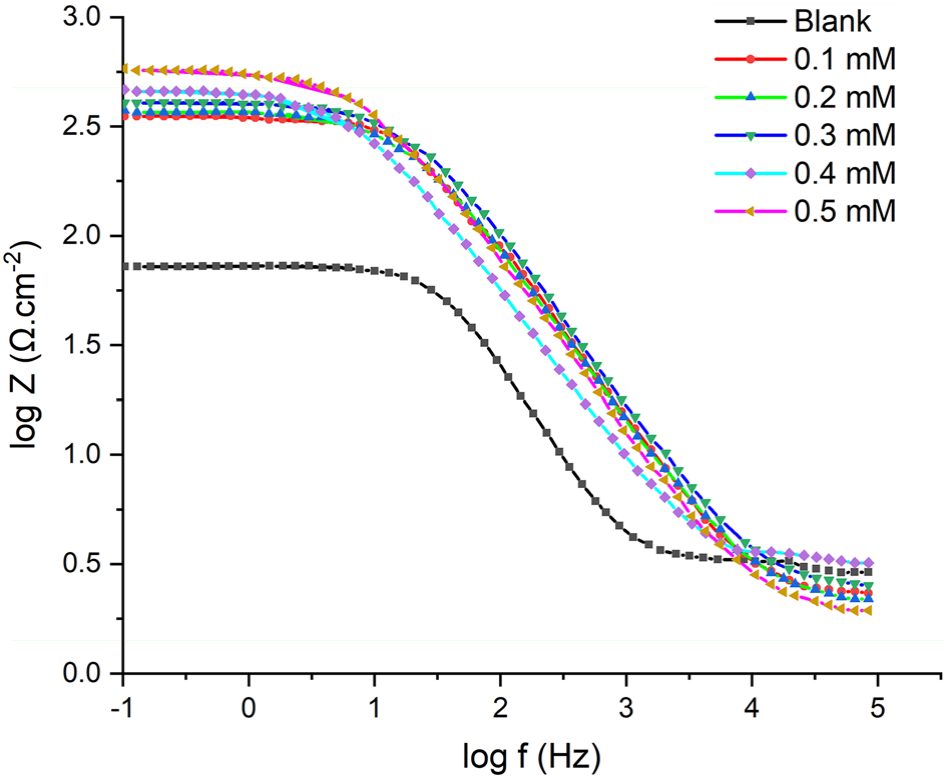
Bode plots of mild steel in 1 M HCl and with different concentrations of 2-TP.

Bode plots are a graphical representation of the frequency response of a system. In electrochemistry, Bode plots are commonly used to analyze the impedance behavior of a system as a function of frequency. They consist of two graphs, one showing the magnitude of the impedance (Bode modulus) and the other showing the phase shift between the applied voltage and the resulting current (Bode phase). These plots can provide valuable information about the electrochemical processes occurring at the electrode surface and the overall behavior of the system. The Bode plots illustrate that a rise in 2-TP concentration causes a broad and significant shift in the Bode modulus impedance, suggesting a slowdown in the corrosion process, as demonstrated in Fig. 10.

The interfacial interaction between 2-TP and the mild steel surface in the acidic solution plays a critical role in inhibiting the corrosion process. The adsorption of 2-TP molecules onto the mild steel surface forms a protective layer that slows down the corrosion process. The adsorption of 2-TP on the mild steel surface occurs through electrostatic interactions, such as hydrogen bonding and van der Waals forces. The presence of nitrogen and sulfur atoms in the 2-TP structure facilitates adsorption on the metal surface by forming strong coordination bonds with iron ions at the metal surface. Moreover, the two aromatic rings in the 2-TP structure provide an extensive π-conjugated system, which interacts with the surface of mild steel by π-π stacking, leading to the formation of an adsorption layer. It is important to note that the adsorption of 2-TP molecules on the mild steel surface may be influenced by the pH of the solution. At low pH, the 2-TP molecules may exist in a protonated form, which can increase their affinity for the negatively charged metal surface. However, as mentioned earlier, it is difficult for 2-TP to be protonated due to the electron-withdrawing effect of the thiophene ring for the nitrogen pair of electrons and steric hindrances. The interaction between 2-TP and the mild steel surface can be studied using electrochemical impedance spectroscopy (EIS). The EIS analysis provides information on the adsorption process, the properties of the protective layer formed on the metal surface, and the corrosion rate of mild steel in the presence of 2-TP. As discussed earlier, the presence of 2-TP significantly increases the polarization resistance and electrolytic resistance of the mild steel sample, indicating the formation of a protective film on the surface of mild steel that acts as a barrier to stop the corrosion reaction. In conclusion, the interfacial interaction between 2-TP and mild steel in the acidic solution is crucial in inhibiting the corrosion process. The adsorption of 2-TP molecules on the mild steel surface occurs through electrostatic interactions, such as hydrogen bonding and van der Waals forces, as well as through coordination bonds with iron ions at the metal surface. The two aromatic rings in the 2-TP structure provide an extensive π-conjugated system, which interacts with the surface of mild steel by π-π stacking, leading to the formation of an adsorption layer. The EIS analysis provides valuable information on the adsorption process, the properties of the protective layer formed on the metal surface, and the corrosion rate of mild steel in the presence of 2-TP.

The interaction between 2-TP and the steel surface can occur via multiple mechanisms. One potential mechanism is adsorption of the 2-TP molecule onto the steel surface through its aromatic rings. The thiophene ring in 2-TP are known to have π-π interactions with metal surfaces, and this can facilitate the adsorption of the inhibitor onto the steel surface. Another potential mechanism is the formation of a protective film on the steel surface via complexation between the nitrogen atom in the pyridine ring and the steel surface. This can lead to the formation of a stable complex that acts as a barrier layer between the steel and the corrosive environment, reducing the corrosion rate.

#### OCR

**Figure 11:**
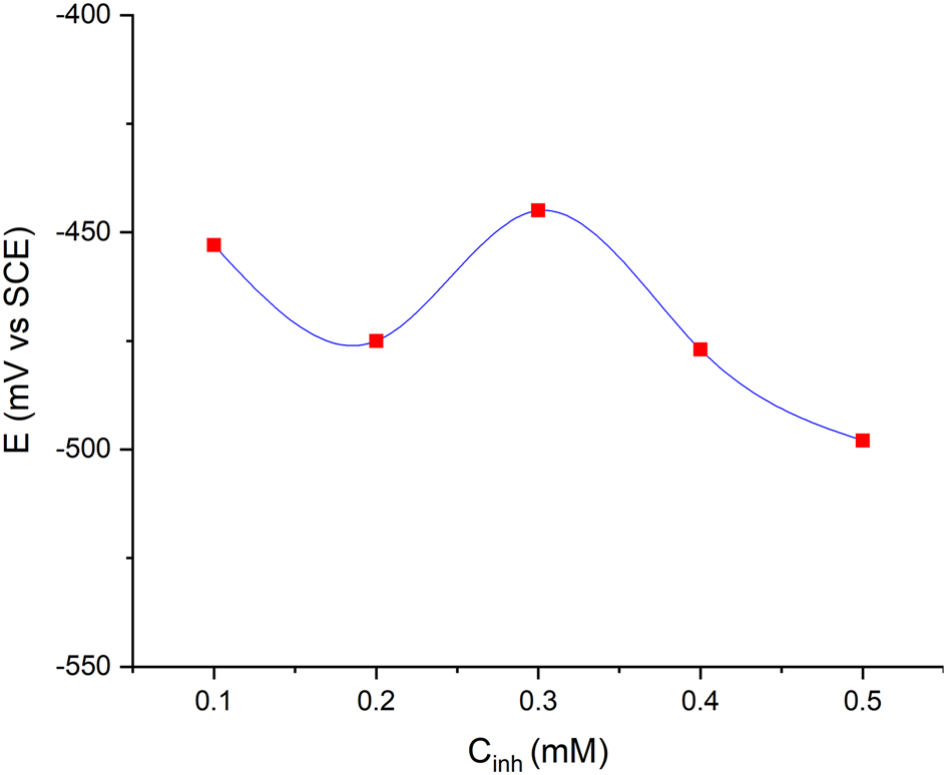
The open circuit potential of mild steel in 1.0 M HCl at 303 K as a function of inhibitor concentration.

Figure 11 shows how the open-circuit potential (OCP) of mild steel in 1.0 M HCl at 303 K changes with the concentration of the 2-TP inhibitor. Inhibition in acidic media typically involves inhibitor molecules adsorbing onto the metal surface, which is often covered with oxide species. The adsorption can result in a more positive surface charge of the mild steel electrode, which is typically negatively charged, due to the presence of the positively charged 2-TP molecules. This change in the OCP suggests that a protective film is forming on the surface of the mild steel electrode.

#### PDP

**Figure 12:**
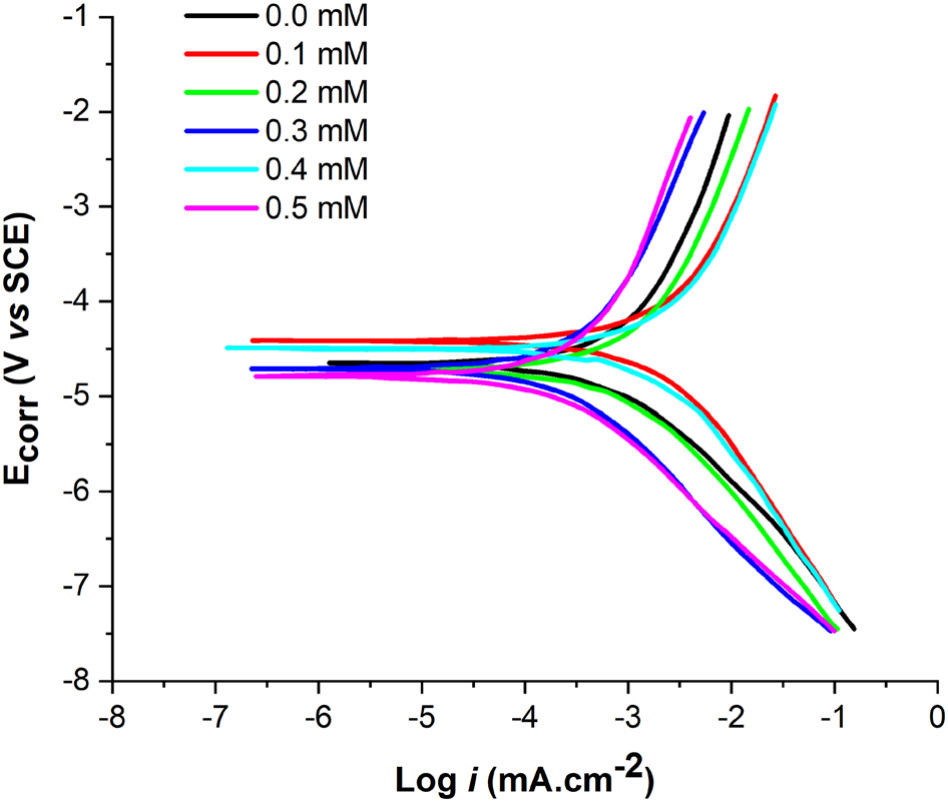
Potentiodynamic Polarization Curves of Mild Steel in Untreated and Treated HCl Solution at 303 K.

A potentiodynamic polarization study offers important insights into the kinetics of the reactions at the anodic and cathodic areas, as well as the inhibition action of corrosion inhibitors based on the corrosion potential^89^. Figure 12 presents polarization plots for mild steel samples in 1 M HCl with and without different concentrations of 2-TP.

Studies have shown that the presence of corrosion inhibitors can suppress the electrochemical reactions on the metal surface, resulting in a decrease in the corrosion current (icorr). This reduction in icorr indicates that corrosion is being inhibited^90^.

The results were obtained by determining the intersection of the anodic and cathodic Tafel lines of the polarization curve at *E*_*CORR*_. The inhibition efficiency was calculated using Eq. (16).

16 $$IE\% = \frac{{i_{corr}^{o} - i_{corr} }}{{i_{corr}^{o} }} \times 100$$

From the polarization plots, it can be seen that as the concentration of 2-TP increases, the corrosion potential shifts towards more negative values, indicating a higher level of corrosion inhibition^91^. This is confirmed by the decrease in the corrosion current, which is shown by the decrease in the slope of the anodic polarization curve^92^. In fact, as can be seen from Fig. 12, both cathodic and anodic polarization curves are displaced due to 2-TP addition. This indicates that the presence of 2-TP affects both the anodic and cathodic reactions, rather than just the cathodic reaction as previously stated. The inhibition efficiency of 2-TP can be calculated from the polarization plots using various methods, such as Tafel slope, corrosion potential, and corrosion current density. The Tafel slope method, based on the slope of the anodic and cathodic polarization curves, provides information about the kinetics of the electrochemical reactions. A decrease in Tafel slope in the presence of 2-TP indicates a slower rate of anodic reactions, resulting in higher inhibition efficiency^93^. Similarly, the corrosion potential can also be used to determine the inhibition efficiency by comparing the corrosion potentials of mild steel samples in 1 M HCl without and with different concentrations of 2-TP. The corrosion current density, which represents the rate of corrosion, can also be used to calculate the inhibition efficiency. A decrease in the corrosion current density in the presence of 2-TP indicates a lower rate of corrosion and higher inhibition efficiency. In conclusion, the potentiodynamic polarization plots provide valuable information about the inhibition efficiency of 2-TP on mild steel in 1 M HCl. The results show that 2-TP is an effective corrosion inhibitor, and its inhibition efficiency increases with the increase in concentration. Table 5 illustrates that the addition of 2-TP resulted in a decrease in the corrosion current density (*i*_*corr*_), indicating that 2-TP has an inhibitory effect on the corrosion of mild steel in the acidic solution at 303 K. This means that the addition of the inhibitor shifts the selected corrosion potentials towards more positive values, suggesting that 2-TP has an inhibitory effect on the corrosion of mild steel at 303 K. The anodic and cathodic processes are altered as different amounts of 2-TP are added, as shown in Fig. 12. If the change in corrosion potential exceeds 85 mV, the inhibitor is categorized as either an anodic or cathodic type inhibitor. However, 2-TP acts as a mixed-type inhibitor, as its highest displacement of 528 mV at 303 K and a significant decrease in i_corr_ for the inhibited system (Table 5) imply that the addition of the tested inhibitor to the acidic solution reduces the anodic solubility of mild steel and slows down the formation of cathodic hydrogen. Furthermore, the results suggest that the addition of 2-TP to the acidic solution at 303 K has a significant impact on the corrosion behavior of mild steel. The inhibitor helps to regulate the balance between the anodic and cathodic reactions, resulting in a decrease in the corrosion rate. The mixed-type inhibitor behavior of 2-TP further highlights its effectiveness in reducing corrosion, as it can target both the anodic and cathodic reactions, resulting in a reduction of both the formation of corrosion products and the hydrogen evolution reaction. This makes 2-TP a valuable tool in controlling corrosion in various applications, particularly in acidic environments.

**Table 5:** Observed corrosion parameters in potentiodynamic polarization measurements.

*C*_*inh*_ (mM)	*E*_*corr*_ (V)	*β*a mV dec^−1^	*− βc* (mV/dec)	*i* (μA/cm^2^)	*IE* (%)
0.0	− 0.475	127.5	258.6	545	61.6
0.1	− 0.511	107.3	198.7	131	73.5
0.2	− 0.509	89.3	181.8	90	75.7
0.3	− 0.541	71.7	193.1	67	83.3
0.4	− 0.498	57.1	157.3	65	88.6
0.5	− 0.528	52.9	131.6	59	90.5

The anodic reaction during the corrosion of metals involves the dissolution of the metal, which results in the formation of metal ions and electrons. The metal ions then migrate into the electrolyte solution, leaving behind electrons that form a layer of negative charge on the metal surface. This process is referred to as oxidation. On the other hand, the cathodic reaction involves the reduction of a species from the electrolyte solution at the metal surface. This reduction reaction consumes the electrons that were released during the anodic reaction. The cathodic reaction can involve various species, such as oxygen, hydrogen ions, or water, depending on the environment in which the corrosion is taking place. In summary, the corrosion process involves both anodic and cathodic reactions that occur simultaneously and continuously, leading to the deterioration of the metal surface. 2-TP is believed to mainly inhibit anodic processes and have a lesser effect on cathodic processes. This is supported by the results of the polarization plots, which show that the cathodic polarization curve remains relatively unchanged in the presence of 2-TP, indicating that the inhibition effect is mainly due to the inhibition of anodic reactions. The inhibition efficiency of 2-TP can be calculated from the polarization plots using various methods, such as Tafel slope, corrosion potential, and corrosion current density. The Tafel slope method, based on the slope of the anodic and cathodic polarization curves, provides information about the kinetics of the electrochemical reactions. A decrease in the Tafel slope in the presence of 2-TP indicates a slower rate of anodic reactions, indicating that 2-TP mainly affects the anodic processes. However, it is important to note that 2-TP may also have some effect on cathodic processes, although this effect is likely to be less significant compared to its effect on anodic processes.

The presence of chloride ions in HCl can have an effect on the performance of 2-TP as a corrosion inhibitor. Chloride ions are known to accelerate the corrosion rate of metals, and they can also compete with the inhibitor molecules for adsorption on the metal surface. Therefore, the presence of high concentrations of chloride ions can reduce the efficiency of 2-TP as a corrosion inhibitor. However, the specific effect of chloride ions on 2-TP would depend on various factors, such as the concentration of the inhibitor and the chloride ions, the nature of the metal, and the pH of the solution.

In conclusion, the results of the study demonstrate that 2-TP has a significant inhibitory effect on the corrosion of mild steel in acidic solutions at 303 K. The inhibitor exhibits mixed-type inhibitor behavior, making it an effective solution for controlling corrosion in acidic environments. Further studies are necessary to determine the mechanism of action of 2-TP and to evaluate its performance under different conditions.

### Scanning electron microscope (SEM)

**Figure 13:**
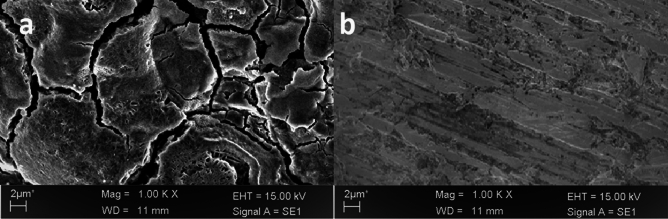
SEM images of the metallic substrate surface after 5 h of exposure in 1 M hydrochloric acid solution (**a**) without and (**b**) with the addition of 2-TP.

The surface morphology of the metallic substrate strip after 5 h of exposure to hydrochloric acid solution with and without 2-TP was analyzed using SEM. The results are presented in Fig. 13a,b. In Fig. 13a, the surface of the metallic substrate exhibited severe corrosion, characterized by sagging and crown features. On the other hand, Fig. 13b shows that the addition of 2-TP to the hydrochloric acid solution resulted in a surface with significantly less corrosion compared to Fig. 13a.

### DFT

#### Calculated parameters

The Inhibition Efficiency Correlation Approach utilizes popular molecular-electronic properties to evaluate the effectiveness of inhibitor molecules. These properties include HOMO and LUMO eigenvalues, HOMO–LUMO gap, electronegativity, chemical hardness, dipole moment, Fukui indices, etc.^94,95^. The approach is based on the premise that these chemical characteristics serve as indicators for reactivity and can predict the direction of inhibitor adsorption bonding^96^. A high HOMO eigenvalue indicates high molecular electron donation, while a low LUMO eigenvalue represents high electron back-donation from surface states to the molecule. A narrow HOMO–LUMO gap means that ELUMO is larger than EHOMO^97^. Theoretical chemistry methods and experimental procedures can be employed to evaluate inhibitor molecules and calculate their efficacy using quantum parameters such as atomic charge, border molecular orbitals, and energy gap. Other factors such as molecular activity, chemical structure, and corrosion inhibitor capacity must also be taken into account^98,99^. The behavior of inhibitors can be understood through their optimal chemical structures and electrochemical behavior in the presence of orbital energies and differences. Frontier molecular orbitals, softness, and hardness are related to the potential of the inhibitor for interaction^100^. Computational chemistry research has been used to determine the efficacy of protection and molecular orbital energy levels of organic compounds^84^. Density Function Theory (DFT), which calculates a molecule's total electron energy based on electron density, has been utilized to study the inhibitory behavior of various sets of corrosion inhibitors. The electronic characteristics were computed and are presented in Table 6.

**Table 6:** Theoretical parameters computed with DFT (d,p) Basis Set using B3LYP Level of 2-TP.

Quantum parameters	2-TP
$I$	8.572 eV
$A$	− 2.887 eV
$E_{HOMO}$	− 8.572 eV
$E_{LUMO}$	2.887 eV
$\Delta E = E_{HOMO} - E_{LUMO}$	− 11.459 eV
$\chi$	2.8925 eV
$\eta$	5.7295
$\sigma$	0.1745
$\Delta N$	0.3584
*µ*	0.8274 D

**Figure 14:**
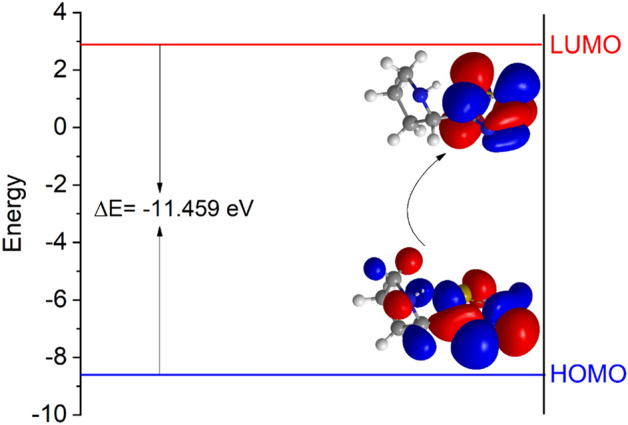
Energy diagram for 2-TP.

The 2-TP inhibition efficacy is linked to the electron-donating potential of EHOMO. By increasing the value of HOMO, the 2-TP inhibition efficacy is also increased. This is because the transfer of charge along the metal surface and initiation of the adsorption mechanism rely on this mechanism. As shown in Fig. 14, the assessed 2-TP is considered to have the greatest inhibitory effectiveness, as it has the highest energy value of HOMO. This high value of HOMO results in a high level of inhibitory effectiveness. The efficacy of electron reception is crucial for ELUMO. A low ELUMO value indicates that inhibitor molecules can find another negative charge on the mild steel surface. The 2-TP molecules have a high LUMO and EHOMO value, indicating their reactiveness as a donor. However, 2-TP molecules with a small EHOMO value decrease metal reactivity and inhibit efficiency^84^.

The 2-TP molecules with the most effective corrosion inhibition, as determined by the EHOMO-ELUMO value, have high softness, low hardness, and a low energy gap. Electronegativity (χ) is also an important factor in inhibiting potency. The inhibiting effects of 2-TP as an iron-inhibitor were studied, revealing that the iron atoms form chemical bonds by gaining electrons from the inhibitor molecules^101^. 2-TP is effective with a low electronegativity value, as indicated by the ΔN value, which shows that the 2-TP molecules transfer more electrons to the iron atoms on the metal surface. Dipole moment (μ) is another factor that may indicate inhibitory efficiency, but previous studies have not shown a significant correlation. The inhibitory efficiency of 2-TP, with a low dipole moment value, suggests a strong coating on the metal surface.

In conclusion, the inhibitory efficiency of 2-TP molecules depends on several factors including the EHOMO-ELUMO value, electronegativity, and dipole moment. 2-TP molecules with a high EHOMO value and low energy gap are highly effective inhibitors due to their softness and low hardness values^102^. Additionally, 2-TP molecules with low electronegativity values provide better performance as they transfer more electrons to the iron atoms on the metal surface. Lastly, 2-TP molecules with a low dipole moment value are believed to provide a stronger coating on the metal surface, contributing to the inhibitory efficiency. These findings provide valuable insights into the development of effective inhibitors for mild steel corrosion protection.

#### Mulliken charges

**Figure 15:**
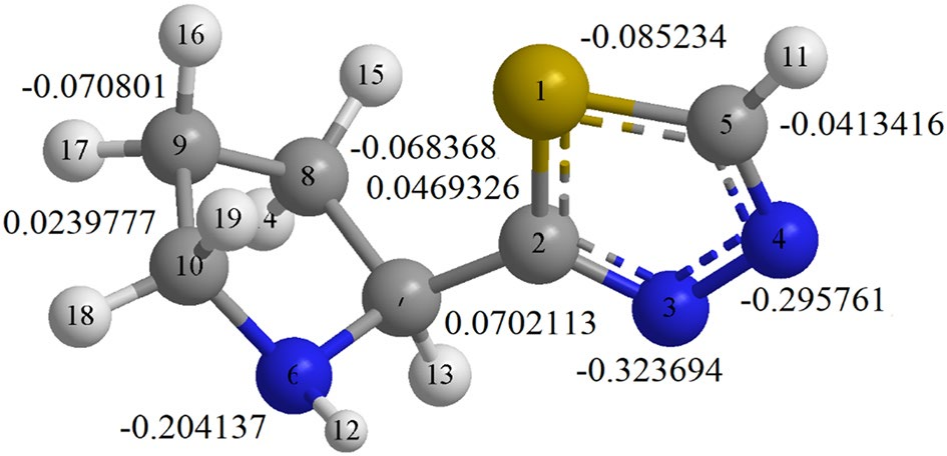
Mulliken charges of 2-TP atoms.

The Mulliken atomic charges of the 2-TP are shown in Fig. 15. The Mulliken charges method is a widely used method to predict the connections between adsorption sites. The heteroatoms in the inhibitor molecules enhance the adsorption ability by donating and accepting electrons. The efficacy of the 2-TP is attributed to the sulfur and nitrogen atoms in the inhibitor molecules^103,104^. As shown in Fig. 15, the 2-TP molecules bond coordinately with the d-orbitals of the iron atoms on the mild steel surface through nitrogen atoms N(3), N(4), and N(6). This leads to the formation of strong coordination bonds between the 2-TP molecules and the mild steel surface, thereby enhancing the corrosion protection provided by the inhibitor. Additionally, the sulfur atom (S(1)) in the 2-TP molecule also contributes to the adsorption process by providing additional electron-donating capacity. The overall charge distribution of the 2-TP molecule also indicates that it is a polar molecule, which enables it to form hydrogen bonds with the mild steel surface, further strengthening the adsorption. In conclusion, the Mulliken atomic charges of the 2-TP molecule offer valuable insights into its adsorption mechanism and effectiveness as a corrosion inhibitor for mild steel surfaces.

### Suggested mechanism

**Figure 16:**
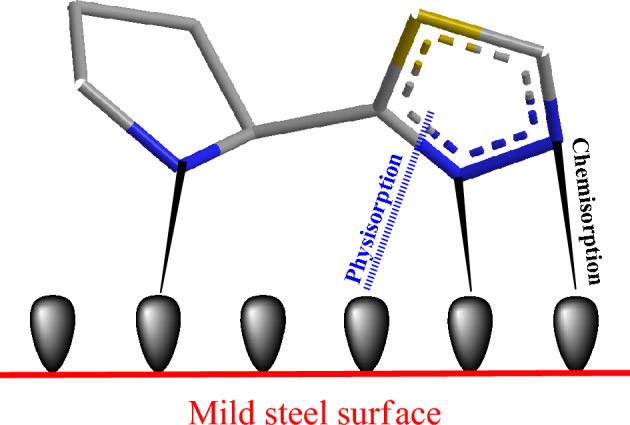
Suggested inhibition mechanism.

The efficiency of corrosion protection on mild steel in acidic conditions can be evaluated based on the molecular structure of the inhibitor and its interaction with the iron atoms on the metal surface. The effectiveness of the inhibitor is influenced by the type of bond formed between the inhibitor and the metal surface (chemisorption) and the number of adsorption sites available. In the case of the 2-TP molecule, all nitrogen atoms serve as adsorption sites and can form coordination bonds with the mild steel surface through the use of unpaired electrons. The nitrogen atoms can also be protonated through physisorption with chloride ions^105^. The presence of unpaired electrons and the inductive effect of the sulfur atom are key factors contributing to the high inhibitive potency of 2-TP. Figure 16 provides a visual representation of the adsorption process of 2-TP molecules on the steel/HCl interface. The free electrons of the nitrogen atoms are transferred to the d-orbitals of the iron atoms, effectively blocking the corrosion process.

This forms a complex with the inhibitor molecule and the iron atom. The adsorption of 2-TP molecules onto the mild steel surface forms a layer of protection, which prevents further corrosion of the metal. This protective layer also slows down the reaction rate of the mild steel and HCl, as the 2-TP molecules act as a barrier between the two. The 2-TP molecule also has a positive effect on the mild steel surface by reducing the electrochemical potential and lowering the corrosive potential. This happens as the adsorption of 2-TP molecules increases the pH of the surrounding environment and thus reduces the concentration of H^+^ ions^106^.

In conclusion, the inhibitor molecule 2-TP exhibits high inhibitive efficiency in protecting mild steel from corrosion in an acidic environment. The molecular structure of the inhibitor and its interaction with the iron atoms of the mild steel surface play a crucial role in the inhibitive efficiency. The 2-TP molecules form a protective layer on the mild steel surface, reducing the reaction rate and lowering the corrosive potential.

## Conclusion

In conclusion, the study demonstrated that 2-(1,3,4-thiadiazole-2-yl)pyrrolidine (2-TP) is an effective inhibitor for mild steel corrosion in 1 M HCl solution. The results from weight loss, potentiodynamic polarization, and electrochemical impedance spectroscopy (EIS) showed that 2-TP adsorbed onto the mild steel surface and formed a protective layer, leading to a reduction in the corrosion rate and increased inhibition efficiency. The study also indicated that temperature had a significant impact on the performance of the inhibitor, with higher temperatures leading to a decrease in inhibition efficiency. The EIS results showed that the presence of 2-TP had a significant impact on the corrosion behavior of mild steel by increasing the polarization resistance and forming a protective film on the metal surface. The potentiodynamic polarization plots showed that the concentration of 2-TP had a significant impact on the corrosion potential and corrosion current, resulting in higher inhibition efficiency. The study highlights the potential of 2-TP as an effective corrosion inhibitor for mild steel in harsh environments.

## Data Availability

The datasets used and/or analysed during the current study available from the corresponding author on reasonable request.

## References

[1] Sharma, B., Verma, A., Prajapati, S. & Sharma, U. K. Synthetic methods, chemistry, and the anticonvulsant activity of thiadiazoles. *Int. J. Med. Chem.* **2013**, 348948. 10.1155/2013/348948 (2013). 25405032 10.1155/2013/348948 PMC4207456

[2] Jain, A. K., Sharma, S., Vaidya, A., Ravichandran, V. & Agrawal, R. K. 1,3,4-thiadiazole and its derivatives: A review on recent progress in biological activities. *Chem. Biol. Drug Des.* **81**(5), 557–576. 10.1111/cbdd.12125 (2013). 23452185 10.1111/cbdd.12125

[3] Salim, R. D. *et al.* Corrosion inhibition of thiadiazole derivative for mild steel in hydrochloric acid solution. *Int. J. Corros. Scale Inhib.* **9**(2), 550–561. 10.17675/2305-6894-2020-9-2-10 (2020).

[4] Annon, I. A. *et al.* Corrosion inhibition of mild steel in hydrochloric acid environment using thiadiazole derivative: Weight loss, thermodynamics, adsorption and computational investigations. *S. Afr. J. Chem. Eng.* **41**, 244–252. 10.1016/j.sajce.2022.06.011 (2022).

[5] Al-Amiery, A. A., Al-Azzawi, W. K. & Isahak, W. N. R. W. Isatin Schiff base is an effective corrosion inhibitor for mild steel in hydrochloric acid solution: Gravimetrical, electrochemical, and computational investigation. *Sci. Rep.* **12**, 17773. 10.1038/s41598-022-22611-4 (2022). 36273029 10.1038/s41598-022-22611-4 PMC9588051

[6] Al-Amiery, A., Kadhum, A., Alobaidy, A. H., Mohamad, A. & Hoon, P. Novel corrosion inhibitor for mild steel in HCl. *Materials* **7**, 662–672 (2014). 28788482 10.3390/ma7020662 PMC5453103

[7] Saha, S. K., Dutta, A., Ghosh, P., Sukul, D. & Banerjee, P. Novel Schiff-base molecules as efficient corrosion inhibitors for mild steel surface in 1 M HCl medium: Experimental and theoretical approach. *Phys. Chem. Chem. Phys.* **18**, 17898–17911. 10.1039/C6CP01993E (2016). 27315235 10.1039/c6cp01993e

[8] Dutta, A., Saha, S. K., Banerjee, P., Patra, A. K. & Sukul, D. Evaluating corrosion inhibition property of some Schiff bases for mild steel in 1 M HCl: Competitive effect of the heteroatom and stereochemical conformation of the molecule. *RSC Adv.* **6**, 74833–74844. 10.1039/C6RA03521C (2016).

[9] Shafek, S. H., Abubshait, S. A., Abubshait, H. A. & Negm, N. A. Antimicrobial potentials and surface activities of novel di-Schiff base nonionic surfactants bearing unsaturated hydrophobic tails. *J. Mol. Liq.* **290**, 110986. 10.1016/j.molliq.2019.110986 (2019).

[10] Saha, S. K., Dutta, A., Ghosh, P., Sukul, D. & Banerjee, P. Adsorption and corrosion inhibition effect of Schiff base molecules on the mild steel surface in 1 M HCl medium: A combined experimental and theoretical approach. *Phys. Chem. Chem. Phys.* **17**, 5679–5690. 10.1039/C4CP05614K (2015). 25623363 10.1039/c4cp05614k

[11] El Basiony, N. M., Elgendy, A., Nady, H., Migahed, M. A. & Zaki, E. G. Adsorption characteristics and inhibition effect of two Schiff base compounds on corrosion of mild steel in 0.5 M HCl solution: Experimental, DFT studies, and Monte Carlo simulation. *RSC Adv.* **9**, 10473–10485. 10.1039/C9RA00397E (2019). 35515280 10.1039/c9ra00397e PMC9062527

[12] Sliem, M. H., El Basiony, N. M., Zaki, E. G., Sharaf, M. A. & Abdullah, A. M. Corrosion inhibition of mild steel in sulfuric acid by a newly synthesized Schiff base: An electrochemical, DFT, and Monte Carlo simulation study. *Electroanalysis* **32**, 3145–3158. 10.1002/elan.202060461 (2020).

[13] Saha, S. K. & Banerjee, P. Introduction of newly synthesized Schiff base molecules as efficient corrosion inhibitors for mild steel in 1 M HCl medium: An experimental, density functional theory and molecular dynamics simulation study. *Mater. Chem. Front.* **2**, 1674–1691. 10.1039/C8QM00162F (2018).

[14] Şafak, S., Duran, B., Yurt, A. & Türkoĝlu, G. Schiff bases as corrosion inhibitor for aluminium in HCl solution. *Corros. Sci.* **54**, 251–259. 10.1016/j.corsci.2011.09.026 (2012).

[15] Fares, M. M. & Bani-Domi, A. Sustainable betalain pigments as eco-friendly film coating over aluminium surface. *J. Mater. Sci.* **56**(24), 13556–13567. 10.1007/s10853-021-06179-4 (2021).

[16] El-Attar, M. & Aazam, E. Redox behavior, spectroscopic investigations, theoretical interpretation and biological effectiveness of some novel prepared bis-azomethine derivatives and their copper(II) complexes. *J. Coord. Chem.* **74**(4–6), 779–803. 10.1080/00958972.2021.1885651 (2021).

[17] El-Lateef, H. M. A., El-Dabea, T., Khalaf, M. M. & Abu-Dief, A. M. Innovation of imine metal chelates as corrosion inhibitors at different media: A collective study. *Int. J. Mol. Sci.* **23**, 9360. 10.3390/ijms23169360 (2022). 36012623 10.3390/ijms23169360 PMC9409127

[18] Hashim, F., Al-Azawi, K., Al-Bghdadi, S. B., Shaker, L. M. & Al-Amiery, A. Experimental and theoretical approach to the corrosion inhibition of mild steel in HCl solution by a newly Coumarin. *Proceedings* **41**, 15. 10.3390/ecsoc-23-06477 (2019).

[19] Mahdi, B. S. *et al.* Corrosion inhibition of mild steel in hydrochloric acid environment using terephthaldehyde based on Schiff base: Gravimetric, thermodynamic, and computational studies. *Molecules* **27**, 4857. 10.3390/molecules27154857 (2022). 35956814 10.3390/molecules27154857 PMC9370009

[20] Al-Amiery, A. A. *et al.* Inhibition of mild steel corrosion in sulfuric acid solution by new Schiff base. *Materials* **7**, 787–804. 10.3390/ma7020787 (2014). 28788488 10.3390/ma7020787 PMC5453085

[21] Betti, N., Al-Amiery, A. A. & Al-Azzawi, W. K. Experimental and quantum chemical investigations on the anticorrosion efficiency of a nicotinehydrazide derivative for mild steel in HCl. *Molecules* **27**, 6254. 10.3390/molecules27196254 (2022). 36234791 10.3390/molecules27196254 PMC9571654

[22] Aziz, I. A. A. *et al.* Weight loss, thermodynamics, SEM, and electrochemical studies on N-2-methylbenzylidene-4-antipyrineamine as an inhibitor for mild steel corrosion in hydrochloric acid. *Lubricants* **10**, 23. 10.3390/lubricants10020023 (2022).

[23] Alkadir Aziz, I. A. *et al.* Insights into corrosion inhibition behavior of a 5-mercapto-1, 2, 4-triazole derivative for mild steel in hydrochloric acid solution: Experimental and DFT studies. *Lubricants* **9**, 122. 10.3390/lubricants9120122 (2021).

[24] Alamiery, A. A., Wan Isahak, W. N. R. & Takriff, M. S. Inhibition of mild steel corrosion by 4-benzyl-1-(4-oxo-4-phenylbutanoyl)thiosemicarbazide: Gravimetrical, adsorption and theoretical studies. *Lubricants* **9**, 93. 10.3390/lubricants9090093 (2021).

[25] Al-Bghdadi, S. B., Hanoon, M. M., Odah, J. F., Shaker, L. M. & Al-Amiery, A. A. Benzylidene as efficient corrosion inhibition of mild steel in acidic solution. *Proceedings* **41**, 27. 10.3390/ecsoc-23-06472 (2019).

[26] Al-Amiery, A. A., Al-Majedy, Y. K., Kadhum, A. A. H. & Mohamad, A. B. New Coumarin derivative as an eco-friendly inhibitor of corrosion of mild steel in acid medium. *Molecules* **20**, 366–383. 10.3390/molecules20010366 (2015). 10.3390/molecules20010366 PMC6272755 25551187

[27] Kadhum, A. A. H. *et al.* Inhibition of mild steel corrosion in hydrochloric acid solution by new Coumarin. *Materials* **7**, 4335–4348. 10.3390/ma7064335 (2014). 28788680 10.3390/ma7064335 PMC5455929

[28] Al-Amiery, A. A., Kadhum, A. A. H., Mohamad, A. B., Musa, A. Y. & Li, C. J. Electrochemical study on newly synthesized chlorocurcumin as an inhibitor for mild steel corrosion in hydrochloric acid. *Materials* **6**, 5466–5477. 10.3390/ma6125466 (2013). 28788402 10.3390/ma6125466 PMC5452761

[29] Junaedi, S., Al-Amiery, A. A., Kadihum, A., Kadhum, A. A. H. & Mohamad, A. B. Inhibition effects of a synthesized novel 4-aminoantipyrine derivative on the corrosion of mild steel in hydrochloric acid solution together with quantum chemical studies. *Int. J. Mol. Sci.* **14**, 11915–11928. 10.3390/ijms140611915 (2013). 23736696 10.3390/ijms140611915 PMC3709763

[30] Al-Amiery, A. A., Kadhum, A. A. H., Mohamad, A. B. & Junaedi, S. A novel hydrazinecarbothioamide as a Potential corrosion inhibitor for mild steel in HCl. *Materials* **6**, 1420–1431. 10.3390/ma6041420 (2013). 28809218 10.3390/ma6041420 PMC5452315

[31] Salman, T. A. *et al.* Effect of 1,3,4-thiadiazole scaffold on the corrosion inhibition of mild steel in acidic medium: An experimental and computational study. *J. Bio. Tribo. Corros.* **5**, 48. 10.1007/s40735-019-0243-7 (2019).

[32] Bentiss, F., Traisnel, M., Vezin, H., Hildebrand, H. F. & Lagrenée, M. 2,5-Bis(4-dimethylaminophenyl)-1,3,4-oxadiazole and 2,5-bis(4-dimethylaminophenyl)-1,3,4-thiadiazole as corrosion inhibitors for mild steel in acidic media. *Corros. Sci.* **46**, 2781–2792 (2004).

[33] Yadav, M., Kumar, S. & Behera, D. Inhibition effect of substituted thiadiazoles on corrosion activity of N80 steel in HCl solution. *J. Metall.* **2013**, 1–14 (2013).

[34] ASTM, G. G 31-72 American Society for Testing and Materials 1990 Philadelphia.

[35] NACE Standard TM 0169/G31-12a. (2012). Standard Guide for Laboratory Immersion Corrosion Testing of Metals.

[36] Umoren, S. A., Solomon, M. M., Obot, I. B. & Suleiman, R. K. Effect of intensifier additives on the performance of butanolic extract of date palm leaves against the corrosion of API 5L X60 carbon steel in 15 wt% HCl solution. *Sustainability* **13**, 5569. 10.3390/su13105569 (2021).

[37] Gaussian 09, Revision D.01, Frisch, M.J.; Trucks, G.W.; Schlegel, H.B.; Scuseria, G.E.; Robb, M.A.; Cheeseman, J.R.; Scalmani, G.; Barone, V.; Mennucci, B.; Petersson, G.A.; Nakatsuji, H.; Caricato, M.; Li, X.; Hratchian, H.P.; Izmaylov, A.F.; Bloino, J.; Zheng, G.; Sonnenberg, J.L.; Hada, M.; Ehara, M.; Toyota, K.; Fukuda, R.; Hasegawa, J.; Ishida, M.; Nakajima, T.; Honda, Y.; Kitao, O.; Nakai, H.; Vreven, T.; Montgomery, Jr., J.A.; Peralta, J.E.; Ogliaro, F.; Bearpark, M.; Heyd, J.J.; Brothers, E.;. Kudin, K.N.; Staroverov, V.N.; Kobayashi, R.; Normand, J.; Raghavachari, K.; Rendell, A.; Burant, J.C.; Iyengar, S.S.; Tomasi, J.; Cossi, M.; Rega, N.; Millam, J.M.; Klene, M.; Knox, J.E.; Cross, J.B.; Bakken, V.; Adamo, C.; Jaramillo, J.; Gomperts, R.; Stratmann, R.E.; Yazyev, O.; Austin, A.J.; Cammi, R.; Pomelli, C.; Ochterski, J.W.; Martin, R.L.; Morokuma, K.; Zakrzewski, V.G.; Voth, G.A.; Salvador, P.; Dannenberg, J.J.; Dapprich, S.; Daniels, A.D.; Farkas, Ö.; Foresman, J.B.; Ortiz, J.V.; Cioslowski, J.; Fox, D.J. Gaussian, Inc., Wallingford CT, 2013.

[38] Koopmans, T. Ordering of wave functions and eigenenergy’s to the individual electrons of an atom. *Physica* **1**, 104–113 (1933).

[39] Saady, A. *et al.* Chemical, electrochemical, quantum, and surface analysis evaluation on the inhibition performance of novel imidazo[4,5-b] pyridine derivatives against mild steel corrosion. *Corros. Sci.* **189**, 109621. 10.1016/j.corsci.2021.109621 (2021).

[40] Mary, Y. S. *et al.* Concentration and solvent dependent SERS, DFT, MD simulations and molecular docking studies of a thioxothiazolidine derivative with antimicrobial properties. *J. Mol. Liq.* **329**, 115582. 10.1016/j.molliq.2021.115582 (2021).

[41] Verma, C. *et al.* Molecular dynamics and Monte Carlo simulations as powerful tools for study of interfacial adsorption behavior of corrosion inhibitors in aqueous phase: A review. *J. Mol. Liq.* **260**, 99–120 (2018).

[42] Zhang, X., Zhang, Y., Su, Y., Wang, X. & Lv, R. Synthesis and corrosion inhibition performance of Mannich bases on mild steel in lactic acid media. *ACS Omega* **7**(36), 32208–32224. 10.1021/acsomega.2c03545 (2022). 36120014 10.1021/acsomega.2c03545 PMC9476531

[43] Alamiery, A. A. Study of corrosion behavior of N’-(2-(2-oxomethylpyrrol-1-yl) ethyl) piperidine for mild steel in the acid environment. *Biointerface Res. Appl. Chem.* **12**(3), 3638–3646 (2022).

[44] Alamiery, A., Mohamad, A. B., Kadhum, A. A. H. & Takriff, M. S. Comparative data on corrosion protection of mild steel in HCl using two new thiazoles. *Data Brief* **40**, 107838 (2022). 35106341 10.1016/j.dib.2022.107838 PMC8784636

[45] Mustafa, A. M. *et al.* Inhibition of mild steel corrosion in hydrochloric acid environment by 1-amino-2-mercapto-5-(4-(pyrrol-1-yl)phenyl)-1,3,4-triazole. *S. Afr. J. Chem. Eng.* **39**, 42–51. 10.1016/j.sajce.2021.11.009 (2022).

[46] Alamiery, A. A. Investigations on corrosion inhibitory effect of newly quinoline derivative on mild steel in HCl solution complemented with antibacterial studies. *Biointerface Res. Appl. Chem.* **12**(2), 1561–1568 (2022).

[47] Alamiery, A. Short report of mild steel corrosion in 0.5 m H_2S_O_4_ by 4-ethyl-1-(4-oxo-4-phenylbutanoyl)thiosemicarbazide. *J. Tribol.* **30**, 90–99 (2021).

[48] Alamiery, A. A., Isahak, W. N. R. W. & Takriff, M. S. Inhibition of mild steel corrosion by 4-benzyl-1-(4-oxo-4-phenylbutanoyl)thiosemicarbazide: Gravimetrical, adsorption and theoretical studies. *Lubricants* **9**(9), 93 (2021).

[49] Dawood, M. A. *et al.* The corrosion inhibition effect of a pyridine derivative for low carbon steel in 1 M HCl medium: Complemented with antibacterial studies. *Int. J. Corros. Scale Inhib.* **10**(4), 1766–1782. 10.17675/2305-6894-2021-10-4-25 (2021).

[50] Alamiery, A. Corrosion inhibition effect of 2-N-phenylamino-5-(3-phenyl-3-oxo-1-propyl)-1,3,4-oxadiazole on mild steel in 1 M hydrochloric acid medium: Insight from gravimetric and DFT investigations. *Mater. Sci. Energy Technol.* **4**, 398–406 (2021).

[51] Alamiery, A. A. Anticorrosion effect of thiosemicarbazide derivative on mild steel in 1 M hydrochloric acid and 0.5 M sulfuric Acid: Gravimetrical and theoretical studies. *Mater. Sci. Energy Technol.* **4**, 263–273 (2021).

[52] Alamiery, A. A., Isahak, W. N. R. W., Aljibori, H. S. S., Al-Asadi, H. A. & Kadhum, A. A. H. Effect of the structure, immersion time and temperature on the corrosion inhibition of 4-pyrrol-1-yl-n-(2,5-dimethyl-pyrrol-1-yl)benzoylamine in 10 m HCl solution. *Int. J. Corros. Scale Inhib.* **10**(2), 700–713. 10.17675/2305-6894-2021-10-2-14 (2021).

[53] Al-Amiery, A. A. *et al.* Experimental and theoretical study on the corrosion inhibition of mild steel by nonanedioic acid derivative in hydrochloric acid solution. *Sci. Rep.* **12**, 4705. 10.1038/s41598-022-08146-8 (2022). 35304485 10.1038/s41598-022-08146-8 PMC8933592

[54] Alamiery, A., Mahmoudi, E. & Allami, T. Corrosion inhibition of low-carbon steel in hydrochloric acid environment using a Schiff base derived from pyrrole: Gravimetric and computational studies. *Int. J. Corros. Scale Inhib.* **10**(2), 749–765. 10.17675/2305-6894-2021-10-2-17 (2021).

[55] Eltmimi, A. J. M. *et al.* Inhibitive effects of a novel efficient Schiff base on mild steel in hydrochloric acid environment. *Int. J. Corros. Scale Inhib.* **10**(2), 634–648. 10.17675/2305-6894-2021-10-2-10 (2021).

[56] Alamiery, A., Shaker, L. M., Allami, T., Kadhum, A. H. & Takriff, M. S. A study of acidic corrosion behavior of Furan-Derived schiff base for mild steel in hydrochloric acid environment: Experimental, and surface investigation. *Mater. Today Proc.* **44**, 2337–2341 (2021).

[57] Al-Baghdadi, S. B., Al-Amiery, A. A., Gaaz, T. S. & Kadhum, A. A. H. Terephthalohydrazide and isophthalo-hydrazide as new corrosion inhibitors for mild steel in hydrochloric acid: Experimental and theoretical approaches. *Koroze a Ochrana Materialu* **65**(1), 12–22 (2021).

[58] Hanoon, M. M., Resen, A. M., Shaker, L. M., Kadhum, A. A. H. & Al-Amiery, A. A. Corrosion investigation of mild steel in aqueous hydrochloric acid environment using n-(Naphthalen-1yl)-1-(4-pyridinyl)methanimine complemented with antibacterial studies. *Biointerface Res. Appl. Chem.* **11**(2), 9735–9743 (2021).

[59] Al-Baghdadi, S., Gaaz, T. S., Al-Adili, A., Al-Amiery, A. A. & Takriff, M. S. Experimental studies on corrosion inhibition performance of acetylthiophene thiosemicarbazone for mild steel in HCl complemented with DFT investigation. *Int. J. Low-Carbon Technol.* **16**(1), 181–188 (2021).

[60] Al-Amiery, A. A. Anti-corrosion performance of 2-isonicotinoyl-n-phenylhydrazinecarbothioamide for mild steel hydrochloric acid solution: Insights from experimental measurements and quantum chemical calculations. *Surf. Rev. Lett.* **28**(3), 2050058 (2021).

[61] Abdulazeez, M. S. *et al.* Corrosion inhibition of low carbon steel in HCl medium using a thiadiazole derivative: Weight loss, DFT studies and antibacterial studies. *Int. J. Corros. Scale Inhib.* **10**(4), 1812–1828. 10.17675/2305-6894-2021-10-4-27 (2021).

[62] Mustafa, A. M. *et al.* Inhibition evaluation of 5-(4-(1H-pyrrol-1-yl)phenyl)-2-mercapto-1,3,4-oxadiazole for the corrosion of mild steel in an acidic environment: Thermodynamic and DFT aspects. *Tribologia Finnish J. Tribol.* **38**(3–4), 39–47. 10.30678/fjt.105330 (2021).

[63] Abdulsahib, Y. M. *et al.* Experimental and theoretical investigations on the inhibition efficiency of N-(2,4-dihydroxytolueneylidene)-4-methylpyridin-2-amine for the corrosion of mild steel in hydrochloric acid. *Int. J. Corros. Scale Inhib.* **10**(3), 885–899. 10.17675/2305-6894-2021-10-3-3 (2021).

[64] Khudhair, A. K. *et al.* Experimental and theoretical investigation on the corrosion inhibitor potential of N-MEH for mild steel in HCl. *Progr. Color Colorants Coat.* **15**(2), 111–122. 10.30509/PCCC.2021.166815.1111 (2021).

[65] Zinad, D. S., Salim, R. D., Betti, N., Shaker, L. M. & AL-Amiery, A. A. Comparative investigations of the corrosion inhibition efficiency of a 1-phenyl- 2-(1-phenylethylidene)hydrazine and its analog against mild steel corrosion in hydrochloric acid solution. *Progr. Color Colorants Coat.* **15**(1), 53–63 (2021).

[66] Salim, R. D., Betti, N., Hanoon, M. & Al-Amiery, A. A. 2-(2,4-Dimethoxybenzylidene)-N-phenylhydrazinecarbothioamide as an efficient corrosion inhibitor for mild steel in acidic environment. *Progr. Color Colorants Coat.* **15**(1), 45–52 (2021).

[67] Al-Amiery, A. A., Shaker, L. M., Kadhum, A. H. & Takriff, M. S. Exploration of furan derivative for application as corrosion inhibitor for mild steel in hydrochloric acid solution: Effect of immersion time and temperature on efficiency. *Mater. Today Proc.* **42**, 2968–2973 (2021).

[68] Resen, A. M. *et al.* Exploration of 8-piperazine-1-ylmethylumbelliferone for application as a corrosion inhibitor for mild steel in hydrochloric acid solution. *Int. J. Corros. Scale Inhib.* **10**(1), 368–387. 10.17675/2305-6894-2021-10-1-21 (2021).

[69] Hanoon, M. M., Resen, A. M., Al-Amiery, A. A., Kadhum, A. A. H. & Takriff, M. S. Theoretical and Experimental Studies on the Corrosion Inhibition Potentials of 2-((6-Methyl-2-Ketoquinolin-3-yl)Methylene) Hydrazinecarbothioamide for Mild Steel in 1 M HCl. *Progr. Color Colorants Coat.* **15**(1), 21–33 (2021).

[70] Hashim, F. G., Salman, T. A., Al-Baghdadi, S. B., Gaaz, T. & Al-Amiery, A. A. Inhibition effect of hydrazine-derived coumarin on a mild steel surface in hydrochloric acid. *Tribologia* **37**(3–4), 45–53 (2020).

[71] Resen, A. M. *et al.* Gravimetrical, theoretical, and surface morphological investigations of corrosion inhibition effect of 4-(benzoimidazole-2-yl) pyridine on mild steel in hydrochloric acid. *Koroze a Ochrana Materialu* **64**(4), 122–130. 10.2478/kom-2020-0018 (2020).

[72] Salman, A. Z., Jawad, Q. A., Ridah, K. S., Shaker, L. M. & Al-Amiery, A. A. Selected BIS-thiadiazole: Synthesis and corrosion inhibition studies on mild steel in HCL environment. *Surf. Rev. Lett.* **27**(12), 2050014 (2020).

[73] Singh, A. K. & Quraishi, M. A. The effect of some bis-thiadiazole derivatives on the corrosion of mild steel in hydrochloric acid. *Corros. Sci.* **52**(4), 1373–1385 (2010).

[74] Singh, A. K. *et al.* Adsorption study of N (-benzo [d] thiazol-2-yl)-1-(thiophene-2-yl) methanimine at mild steel/aqueous H2SO4 interface. *Surf. Interfaces* **33**, 102169 (2022).

[75] Chugh, B. *et al.* Comparative investigation of corrosion-mitigating behavior of thiadiazole-derived bis-schiff bases for mild steel in acid medium: Experimental, theoretical, and surface study. *ACS Omega* **5**(23), 13503–13520 (2020). 32566815 10.1021/acsomega.9b04274 PMC7301369

[76] Solomon, M. M., Umoren, S. A., Quraishi, M. A., Tripathy, D. B. & Abai, E. J. Effect of akyl chain length, flow, and temperature on the corrosion inhibition of carbon steel in a simulated acidizing environment by an imidazoline-based inhibitor. *J. Pet. Sci. Eng.* **187**, 106801. 10.1016/j.petrol.2019.106801 (2020).

[77] Farag, A. A. *et al.* The inhibition performance of morpholinium derivatives on corrosion behavior of carbon steel in the acidized formation water: Theoretical, experimental and biocidal evaluations. *J. Mol. Liq.* **341**, 117348. 10.1016/j.molliq.2021.117348 (2021).

[78] Kokalj, A. Molecular modeling of organic corrosion inhibitors: Calculations, pitfalls, and conceptualization of molecule–surface bonding. *Corros. Sci.* **193**, 109650. 10.1016/j.corsci.2021.109650 (2021).

[79] Mobin, M., Rizvi, M., Olasunkanmi, L. O. & Ebenso, E. E. Biopolymer from Tragacanth gum as a green corrosion inhibitor for carbon steel in 1 M HCl solution. *ACS Omega* **2**, 3997–4008 (2017). 31457703 10.1021/acsomega.7b00436 PMC6641194

[80] Eddy, N. O. & Ebenso, E. E. Adsorption and inhibitive properties of ethanol extracts of *Musa sapientum* peels as a green corrosion inhibitor for mild steel in H2SO4. *Afr. J. Pure Appl. Chem.* **2**(6), 046–054 (2008).

[81] Rani, P. D. & Selvaraj, S. *Emblica officinalis* (AMILA) leaves extract as corrosion inhibitor for copper and its alloy (Cu- 272N) in natural sea water. *Arch. Appl. Sci. Res.* **2**(6), 140–150 (2010).

[82] Iloamaeke, I. M., Egwuatu, C. I., Umeobika, U. C. & Edike, H. Corrosion inhibition and adsorption studies of ethanol extract of Senna alata for mild steel in 2.0M H_2_SO_4_ solution. *Int. J. Mater. Chem. Phys.* **1**(3), 295–299 (2015).

[83] Meroufel, B., Benali, O., Benyahia, M., Benmoussa, Y. & Zenasni, M. A. Adsorptive removal of anionic dye from aqueous solutions by algerian kaolin: Characteristics, isotherm, kinetic and thermodynamic studies. *J. Mater. Environ. Sci.* **3**(4), 482–491 (2013).

[84] Deng, S., Li, X. & Xie, X. Hydroxymethyl urea and 1,3- bis(hydroxymethyl) urea as corrosion inhibitors for steel in HCl solution. *Corros. Sci.* **80**, 276–289 (2014).

[85] Espinoza Vázquez, A. *et al.* Corrosion inhibition assessment on API 5L X70 steel by preussomerin G immersed in saline and saline acetic. *J. Adhes. Sci. Technol.* **35**, 873–899. 10.1080/01694243.2020.1826828 (2021).

[86] Pang, L. *et al.* Inhibition performance of benzimidazole derivatives with different heteroatoms on the under-deposit corrosion of carbon steel in CO_2_-saturated solution. *Corros. Sci.* **192**, 109841. 10.1016/j.corsci.2021.109841 (2021).

[87] Zhang, G. A., Liu, D., Li, Y. Z. & Guo, X. P. Corrosion behavior of N80 carbon steel in formation water under dynamic supercritical CO_2_ condition. *Corros. Sci.* **120**, 107–120. 10.1016/j.corsci.2017.02.012 (2017).

[88] Varvara, S. *et al.* Multiscale electrochemical analysis of the corrosion control of bronze in simulated acid rain by horse-chestnut extract as green inhibitor. *Corros. Sci.* **165**, 108381. 10.1016/j.corsci.2019.108381 (2020).

[89] Pan, J., Thierry, D. & Leygraf, C. Electrochemical impedance spectroscopy study of the passive oxide film on titanium for implant application. *Electrochim. Acta* **41**, 1143–1153. 10.1016/0013-4686(95)00465-3 (1996).

[90] Onyeachu, I. B., Obot, I. B. & Adesina, A. Y. Green corrosion inhibitor for oilfield application II: The time–evolution effect on the sweet corrosion of API X60 steel in synthetic brine and the inhibition performance of 2-(2-pyridyl) benzimidazole under turbulent hydrodynamics. *Corros. Sci.* **168**, 108589. 10.1016/j.corsci.2020.108589 (2020).

[91] Corrales Luna, M. *et al.* Study of corrosion behavior of API 5L X52 steel in sulfuric acid in the presence of ionic liquid 1-ethyl 3-methylimidazolium thiocyanate as corrosion inhibitor. *J. Mol. Liq.* **289**, 111106. 10.1016/j.molliq.2019.111106 (2019).

[92] Salman, M. *et al.* Chromeno naphthyridines based heterocyclic compounds as novel acidizing corrosion inhibitors: Experimental, surface and computational study. *J. Mol. Liq.* **322**, 114825. 10.1016/j.molliq.2020.114825 (2021).

[93] Chauhan, L. R. & Gunasekaran, G. Corrosion inhibition of mild steel by plant extract in dilute HCl medium. *Corros. Sci.* **49**(3), 1143–1161. 10.1016/j.corsci.2006.08.012 (2007).

[94] Behpour, M. *et al.* Electrochemical and theoretical investigation on the corrosion inhibition of mild steel by thiosalicylaldehyde derivatives in hydrochloric acid solution. *Corros. Sci.* **50**, 2172–2181. 10.1016/j.corsci.2008.06.020 (2008).

[95] Zarrok, H. *et al.* Quantum chemical study on the corrosion inhibition of some bipyrazoles. *Res. J. Pharm. Biol. Chem. Sci.* **6**, 1853–1860 (2015).

[96] Gece, G. The use of quantum chemical methods in corrosion inhibitor studies. *Corros. Sci.* **50**, 2981–2992 (2008).

[97] Obot, I. B., Macdonald, D. D. & Gasem, Z. M. Density functional theory (DFT) as a powerful tool for designing new organic corrosion inhibitors: Part 1: An overview. *Corros. Sci.* **99**, 1–30 (2015).

[98] Huong, D. Q., Duong, T. & Nam, P. C. Experimental and theoretical study of corrosion inhibition performance of N-phenylthiourea for mild steel in hydrochloric acid and sodium chloride solution. *J. Mol. Model.* **25**, 204 (2019). 31250118 10.1007/s00894-019-4084-6

[99] Camacho-Mendoza, R. L. *et al.* Density functional theory and electrochemical studies: Structure-efficiency relationship on corrosion inhibition. *J. Chem. Inf. Model.* **55**, 2391–2402 (2015). 26505207 10.1021/acs.jcim.5b00385

[100] Khalil, N. Quantum chemical approach of corrosion inhibition. *Electrochim. Acta* **48**, 2635–2640 (2003).

[101] Ebenso, E. E., Isabirye, D. A. & Eddy, N. O. Adsorption and quantum chemical studies on the inhibition potentials of some thiosemicarbazides for the corrosion of mild steel in acidic medium. *Int. J. Mol. Sci.* **11**, 2473–2498 (2010). 20640164 10.3390/ijms11062473 PMC2904928

[102] Parr, R. G. & Pearson, R. G. Absolute hardness: Companion parameter to absolute electronegativity. *J. Am. Chem. Soc.* **105**, 7512–7516 (1983).

[103] Singh, P., Kumar, M., Quraishi, M. A., Haque, J. & Singh, G. Bispyranopyrazoles as green corrosion inhibitors for mild steel in hydrochloric acid: Experimental and theoretical approach. *ACS Omega* **3**, 11151–11162 (2018). 31459224 10.1021/acsomega.8b01300 PMC6645101

[104] Ibrahim, T., Gomes, E., Obot, I. B., Khamis, M. & Abou Zour, M. Corrosion inhibition of mild steel by Calotropisprocera leaves extract in a CO2 saturated sodium chloride solution. *J. Adhes. Sci. Technol.* **30**, 2523–2543 (2016).

[105] Yadav, D. K., Chauhan, D. S., Ahamad, I. & Quraishi, M. A. Electrochemical behavior of steel/acid interface: Adsorption and inhibition effect of oligomeric aniline. *RSC Adv.* **3**, 632–646 (2013).

[106] Ansari, K. R. & Quraishi, M. A. Singh, Pyridine derivatives as corrosion inhibitors for N80 steel in 15% HCl: Electrochemical, surface and quantum chemical studies. *Measurement* **76**, 136–147 (2015).

