# Author Correction: Multi-method evaluation of a 2-(1,3,4-thiadiazole-2-yl)pyrrolidine corrosion inhibitor for mild steel in HCl: combining gravimetric, electrochemical, and DFT approaches

**DOI:** 10.1038/s41598-024-83688-7

**Published:** 2025-01-03

**Authors:** Ahmed Al-Amiery, Wan Nor Roslam Wan Isahak, Waleed Khalid Al-Azzawi

**Affiliations:** 1 Department of Chemical and Process Engineering, Faculty of Engineering and Built Environment, Universiti Kebangsaan Malaysia, Bangi, Malaysia; 2 University of Technology-Iraq, Energy and Renewable Energies Technology Center, Bagdad, Iraq; 3 Al-Farahidi University, Baghdad, 10001 Iraq

Correction to: *Scientific Reports* 10.1038/s41598-023-36252-8, published online 16 June 2023

The original version of this Article contained an error in the Results section under the subheading ‘Electrochemical corrosion measurements’, ‘PDP’. During the revision of the Article, the comment “Thank you for bringing this to my attention.” inadvertently remained in the text.

As a result,

"This indicates that the presence of 2-TP affects both the anodic and cathodic reactions, rather than just the cathodic reaction as previously stated. Thank you for bringing this to my attention. The inhibition efficiency of 2-TP can be calculated from the polarization plots using various methods, such as Tafel slope, corrosion potential, and corrosion current density."

now reads:

"This indicates that the presence of 2-TP affects both the anodic and cathodic reactions, rather than just the cathodic reaction as previously stated. The inhibition efficiency of 2-TP can be calculated from the polarization plots using various methods, such as Tafel slope, corrosion potential, and corrosion current density."

The original Article has been corrected.

